# DANCE: Step‐by‐Step Protocol for Automated Quantification of *Drosophila* Aggression and Courtship

**DOI:** 10.1002/cpz1.70511

**Published:** 2026-11-14

**Authors:** R. Sai Prathap Yadav, Paulami Dey, Pavan Agrawal

**Affiliations:** ^1^ Department of Anatomy, Kasturba Medical College Manipal Academy of Higher Education Manipal India

**Keywords:** aggression, automated behavioral quantification, courtship, *Drosophila*, JAABA

## Abstract

Aggression and courtship are widely studied social behaviors in *Drosophila melanogaster*, but their accurate quantification remains a practical obstacle. Manual scoring of these behaviors is time consuming, prone to inter‐observer variability, and difficult to scale, while existing automated tools often require expensive hardware or specialized skills. This article provides step‐by‐step protocols for implementing DANCE (*Drosophila* Aggression and Courtship Evaluator), a low‐cost platform that combines supervised machine learning classifiers with recording hardware built from repurposed medicine blister packs, acrylic sheets, and consumer smartphones. The protocols describe how to construct the aggression and courtship arenas, acquire standardized videos using a smartphone over a tablet backlight, and process recordings through an automated pipeline that tracks individual flies in FlyTracker and scores behavior using six pretrained JAABA classifiers, one for aggressive lunges and five for the stages of male courtship. Additional protocols describe how to train new classifiers, validate classifier performance against manual annotation, and add an optogenetic stimulation module. Each protocol specifies the required materials, software versions, parameter settings, and expected outputs, so that a user with no prior experience in automated behavioral analysis can generate per‐dyad behavioral measurements suitable for statistical comparison. All classifiers, analysis scripts, and worked examples are available at https://github.com/agrawallab/DANCE. The platform was originally described and validated by Yadav et al. (2025). © 2026 The Author(s). *Current Protocols* published by Wiley Periodicals LLC.

**Basic Protocol 1**: Construction and use of DANCE hardware for recording aggression in *Drosophila*

**Support Protocol 1**: Preparation of *Drosophila* for aggression and courtship assays

**Support Protocol 2**: Preparation of aggression food and arena coating

**Support Protocol 3**: Standardized video acquisition for DANCE recordings

**Basic Protocol 2**: Construction and use of DANCE hardware for recording courtship behaviors in *Drosophila*

**Basic Protocol 3**: Automated quantification of aggression and courtship behaviors

**Support Protocol 4**: Animal tracking using Caltech FlyTracker

**Support Protocol 5**: Score file generation using JAABA classifiers

**Alternate Protocol 1**: Training custom JAABA classifiers

**Alternate Protocol 2**: Ground‐truth annotation and classifier validation

**Alternate Protocol 3**: Integration of an optogenetics module

## INTRODUCTION

Aggression and courtship in *Drosophila melanogaster* have been used for decades to study the genetic and neural basis of social behavior. Progress in this area depends on measuring these behaviors reliably, but quantification remains a bottleneck. Manually scoring aggressive lunges and courtship behaviors such as wing extension and copulation is time consuming, prone to inter‐observer variability, and difficult to scale to the number of animals that a genetic screen requires. This article describes how to set up and run DANCE, a low‐cost platform that automates the scoring of *Drosophila* aggression and courtship and can be assembled and operated by a single laboratory without specialized engineering or programming support.

Existing automated tools for *Drosophila* social behavior fall broadly into two groups, each with practical limitations. Rule‐based tools such as CADABRA (Dankert et al., 2009) and MateBook (Ribeiro et al., 2018) apply fixed definitions of each behavior. These definitions do not adapt well to differences between individuals, genotypes, or recording setups, and they can miss or misclassify behavioral events that manual ground‐truth scoring would capture (Simon and Heberlein, 2020). Classifiers built on supervised machine learning using JAABA (Kabra et al., 2013) are more adaptable, but they are often not shared publicly, and the pipelines that use them frequently depend on custom‐machined arenas or machine‐vision cameras that are costly to obtain (Chowdhury et al., 2021; Leng et al., 2020). As a result, automated behavioral scoring has remained inaccessible to many resource‐constrained laboratories.

DANCE was developed to address these limitations. The hardware is built from inexpensive, widely available materials and a consumer smartphone, and the analysis uses freely available classifiers that quantify aggressive lunges and five distinct courtship behaviors. The platform and its validation against existing methods are described in full in the original research article (Yadav et al., 2025). The present article provides the procedures needed to build the platform and apply it to new experiments.

The protocols follow the experimental workflow. Basic Protocol 1, together with Support Protocols 1, 2, and 3, covers construction of the aggression arena, fly husbandry, food and arena preparation, and standardized video acquisition. Basic Protocol 2 covers the construction of the courtship arena. Basic Protocol 3 describes the complete analysis pipeline, supported by Support Protocol 4 for fly tracking in FlyTracker and Support Protocol 5 for behavioral scoring with pretrained JAABA classifiers. Alternate Protocols 1 and 2 describe how to train and validate new classifiers, and Alternate Protocol 3 describes how to add an optogenetic stimulation module.

## Basic Protocol 1 CONSTRUCTION AND USE OF DANCE HARDWARE FOR RECORDING AGGRESSION IN *DROSOPHILA*

This protocol describes the step‐by‐step assembly of the DANCE recording platform for aggression assays. The hardware uses repurposed transparent medicine blister packs, mounted on acrylic base plates over an apple‐juice agar food surface, as behavioral arenas. Arena walls and ceilings are coated with Sigmacote to prevent wall‐climbing. Complete Support Protocol 1 (fly preparation) 6 days in advance, Support Protocol 2 (food preparation and arena coating) the day before recording, and Support Protocol 3 (video acquisition setup) before loading flies. A video demonstration of DANCE aggression hardware assembly and recording is available as Figure 6—video 2 of Yadav et al. (2025; https://elifesciences.org/articles/105465/figures#fig6video2), and it is recommended that first‐time users view this before beginning assembly. An overview of the DANCE hardware components and recording setup is shown in Figure 1. Arena assembly for one 8‐well session takes ∼5 min.

**Figure 1:**
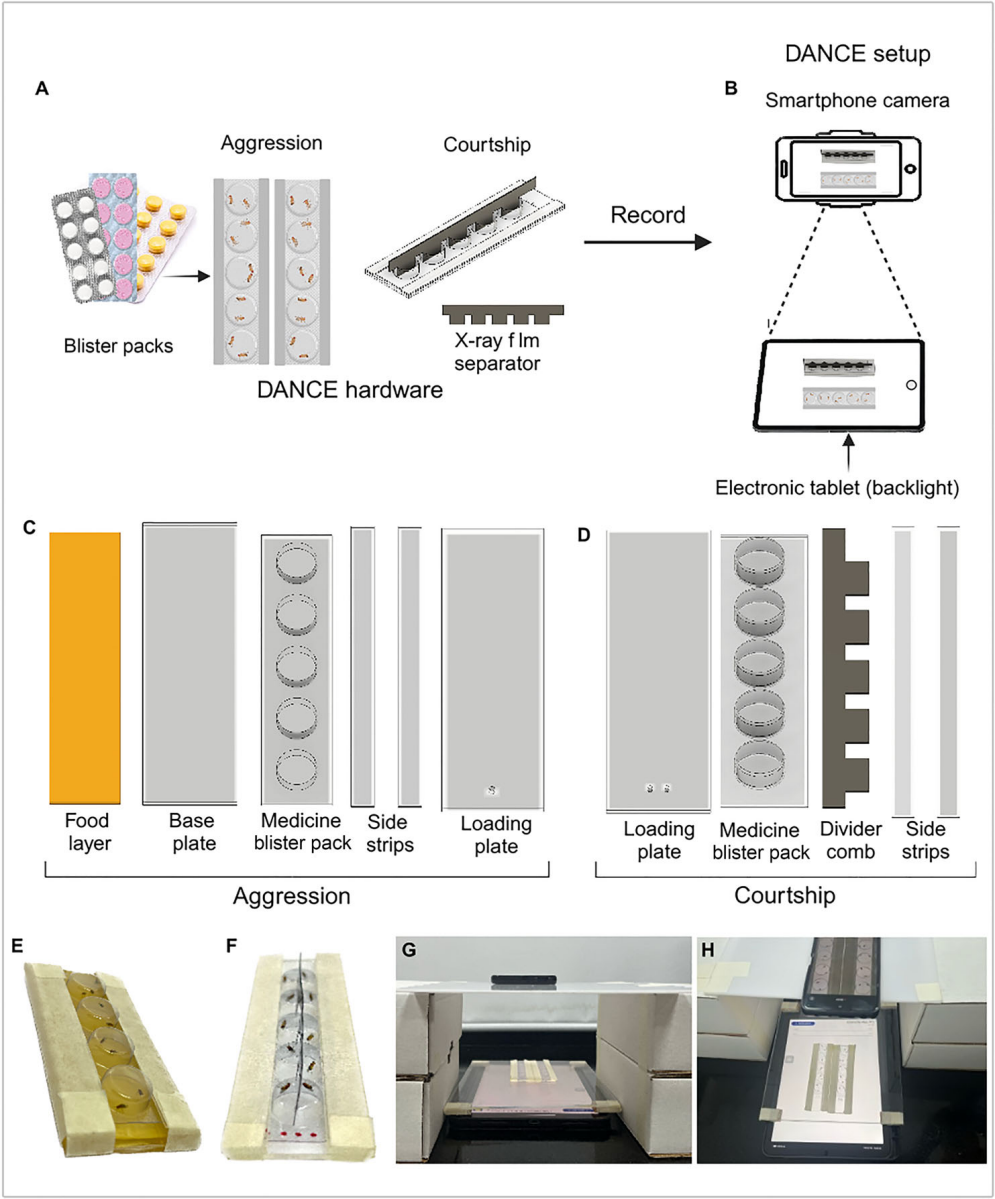
DANCE hardware and recording setup for *Drosophila* aggression and courtship assays. (**A**) Schematic overview of the DANCE (*Drosophila* Aggression and Courtship Evaluator) hardware. Aggression arenas are constructed from repurposed medicine blister packs; courtship arenas use the same format with the addition of X‐ray film separator combs. (**B**) Behavioral videos are acquired using a smartphone camera positioned above the DANCE hardware. The arenas are illuminated from below using an electronic tablet that serves as a uniform backlight source, enabling high‐contrast video acquisition suitable for automated tracking. (**C**) Exploded view of the aggression assay chamber showing the food layer, base plate, medicine blister packs containing circular arenas, side strips, and loading plate. (**D**) Exploded view of the courtship assay chamber showing the loading plate, medicine blister packs, divider comb, and side strips. (**E**) Photograph of an assembled aggression chamber with food and flies. (**F**) Photograph of an assembled courtship chamber with flies separated by a divider comb. (**G**) Side view of recording setup, showing DANCE chambers positioned on an illuminated electronic tablet between supporting structures to maintain a fixed camera‐to‐arena distance during video acquisition. (**H**) Top view of smartphone‐based imaging platform used for behavioral recording. The DANCE chamber is positioned above the illuminated tablet, separated by a 4‐mm acrylic spacer. Videos are recorded from above using a smartphone camera.

### Materials

Male flies, some group housed and some singly housed for 6 days (Support Protocol 1)
Apple‐juice agar food plates (see recipe and Support Protocol 2)

Transparent circular medicine blister packs (standard aggression arena: 13 mm diameter × 5.5 mm height; alternatives: 17 mm × 4 mm and 21 mm × 8 mm), coated with Sigmacote (see Support Protocol 2)
2‐mm‐thickness acrylic food plate
Razor blade
1‐mm‐thickness acrylic side spacers
Paper tape
2‐mm‐thickness acrylic base plate with 2‐mm loading holes
Drill machine (if loading holes are not pre‐drilled)
Mouth aspirator
Electronic tablet or smartphone displaying a white screen light application (for backlight)
Transparent acrylic sheet
4‐mm acrylic spacers
Android or iPhone smartphone camera (for recording; see Support Protocol 3 for tested models and format requirements)

*NOTE*: Prepare food plates and coat blister‐pack arenas with Sigmacote at least 12‐16 hr before beginning this protocol. See Support Protocol 2 for food preparation and arena coating instructions.

#### Preparing fly behavioral arenas

1 Select transparent medicine blister packs with circular wells to serve as behavioral arenas. The standard arena for aggression recording measures 13 mm diameter × 5.5 mm height. Alternative dimensions of 17 mm × 4 mm or 21 mm × 8 mm may be used for fly strains for which a different interaction density is required.
2 Verify that the arena walls and ceilings have been coated with Sigmacote and allowed to dry overnight (see Support Protocol 2, steps 11‐16). Confirm coating effectiveness by introducing a single fly into one coated well for 1‐2 min before the experiment: a properly coated arena will keep the fly on the food surface without wall‐climbing. Discard arenas in which wall‐climbing is observed.
*CAUTION*: All Sigmacote applications must be performed in a fume hood. Sigmacote (SL2) contains chlorotrimethylsilane in a heptane/xylene carrier and is flammable and a respiratory irritant. See Support Protocol 2 for full coating procedure.
3 Take the apple‐juice agar food plate prepared in advance, cut it to the appropriate size using a razor blade, and place on a flat surface with the food surface facing upward.
4 Secure the food plate using paper tape and 1‐mm acrylic side spacers.
5 Similarly, secure the loading base plate containing the medicine blister pack arenas using paper tape and acrylic spacers to ensure proper alignment and stability.

#### Loading flies into arenas

6 Load flies into the assembled arenas (loading base plate with medicine blister pack arenas) using a mouth aspirator. Transfer exactly two males from the same housing condition into each well through the 2‐mm loading hole, one at a time; confirm that the first male has entered the well before loading the second. Keep the loading hole covered by sliding the loading plate over the adjacent chamber between transfers.
*IMPORTANT NOTE*: Do not anesthetize males before loading—transfer males directly from their housing vials into the arena by mouth aspirator, without CO_2_ exposure. Anesthesia on the day of the assay will suppress aggression and render the recording unusable.
  Some males should have been individually housed (experimental group) and others group housed (control group) for 6 days before this step (see Support Protocol 1, steps 5a and 5b). Do not load flies that appear lethargic or damaged.
7 Confirm that both males are inside the well, and seal the loading hole with a strip of paper tape, pressing firmly.
8 Apply a strip of paper tape along one end of the food plate.
  This tape protects the food surface from damage when the blister‐pack arenas are slid into position during assembly.
9 Align the 2‐mm acrylic loading base plate with the flies in medicine blister pack arenas over the food plate. Join the two components with paper tape along the edges, ensuring that the food surface is level and aligned evenly with the loading base plate with medicine blister pack arenas.
10 Slide the coated blister‐pack arenas with flies over the food plate from the paper‐tape end, guiding the wells to align with the food surface. Slide slowly to avoid displacing the food.
*IMPORTANT NOTE*: Always slide the arenas from the paper‐tape end. The tape at the leading edge prevents the blister pack from catching and tearing the food surface during assembly.
11 Once the arenas are in position over the food plate, seal all edges with paper tape to prevent fly escape. Press tape firmly along all seams including between adjacent wells.
12 Verify that all wells are correctly positioned over the food surface by looking through the transparent blister‐pack ceiling. The food surface should be visible at the bottom of each well.
13 Allow flies to acclimatize in the sealed arena for 5 min. Place the loaded arena on the backlight setup (step 14) immediately after sealing. Keep the backlight on during acclimatization. Do not begin recording until the full acclimatization period has elapsed.
*IMPORTANT NOTE*: Acclimatization in the sealed arena is required before recording for the reliability of behavioral output. The 5‐min acclimatization period is not counted as part of the 20‐min recording window.

#### Recording fly behavior

14 Place the assembled arena on an electronic tablet or smartphone displaying a white screen light application at full brightness. This serves as the backlight illumination source.
15 Place a transparent acrylic sheet with 4‐mm spacers on top of the backlight device. Place the arena on top of this sheet.
  The 4‐mm air gap allows heat dissipation and prevents the arenas from overheating.
*IMPORTANT NOTE*: Never place arenas directly on the tablet or smartphone screen. The 4‐mm spacer is essential for preventing heat buildup, which suppresses behavioral output.
16 Position the smartphone camera directly above the arena in a fixed, vertical orientation. Configure recording parameters and begin recording as described in Support Protocol 3. Record for 20 min for aggression assays.

#### Time considerations

Hardware assembly (excluding overnight drying steps completed the day before) takes ∼5 min for an eight‐well session. Fly loading takes ∼3 min for eight wells. Acclimatization takes 5 min. Recording takes 20 min. Total time from assembly start to end of recording: ∼45 min. With practice, fly loading and assembly time decreases substantially from the estimates above. For higher throughput, multiple 8‐well arenas can be placed under separate smartphone cameras simultaneously, with two to four cameras running in parallel in a single session.

#### Notes

• Blister‐pack arenas are reusable for ∼30‐40 aggression sessions when washed in 0.05% Alcojet solution and air‐dried (see Recipes). Do not use heat to dry arenas.
• Re‐coat arenas with Sigmacote after every three to five uses or when wall‐climbing is observed.
• Ensure all gaps are sealed before loading. Any unsealed gap will result in fly escape.

## Support Protocol 1 PREPARATION OF *DROSOPHILA* FOR AGGRESSION AND COURTSHIP ASSAYS

This protocol describes the rearing, collection, and housing conditions required for male *Drosophila melanogaster* used in DANCE aggression and courtship assays, and the preparation of female flies for courtship assays. Complete this protocol in advance of all behavioral experiments. Males must be collected and housed 6 days before the planned assay date. Steps 1‐9 apply to both aggression and courtship assays; steps 10a‐13a and 10b‐11b apply to courtship assays only.

### Materials

Fly stocks and food
Canton‐S (CS) wild‐type *Drosophila melanogaster*
Standard *Drosophila* food vials: group housing (90 mm length × 25 mm diameter); individual housing (70 mm length × 10 mm diameter)
Standard *Drosophila* food (cornmeal‐yeast‐agar or equivalent)

Fly incubator set to 25°C and 12‐hr/12‐hr light/dark cycle
Mouth aspirator
CO_2_ delivery system
CO_2_‐based fly sorting pad
Paintbrush
Stereomicroscope
Microdissection scissors (for decapitation of virgin females, step 11b)

#### Fly rearing and maintenance

1 Rear all fly stocks at 25°C, 65% relative humidity, under a 12‐hr/12‐hr light/dark cycle. Zeitgeber time zero (ZT0) is defined as the onset of the light phase. Record ZT0 for the incubator and use it as the reference for all assay scheduling in steps 7‐9.
2 Maintain Canton‐S stocks on standard *Drosophila* food. Transfer stocks to fresh food vials every 2‐3 weeks to avoid overcrowding. Do not use stocks that have been reared at nonstandard temperatures or under constant light.

#### Collection of male flies for aggression and courtship assays

3 On the day of collection (6 days before the planned assay), identify vials containing newly eclosed flies. Newly eclosed males are recognizable by their pale, unhardened cuticle, which darkens within several hours after eclosion. Collect males within 24 hr of eclosion.
  Collecting males outside the 24‐hr post‐eclosion window introduces age heterogeneity within a cohort. Because male aggression levels increase with age and then plateau, even a 12‐hr difference in collection time can increase variance within groups.
4 Anesthetize flies briefly on a CO_2_ pad. Using a paintbrush or feather, sex‐sort flies under a stereomicroscope, transferring males to a holding vial with standard food.

#### Housing of male flies before assays

5a *For group‐housed (GH) control males*: Transfer groups of 20 male flies to standard group‐housing vials (90 mm × 25 mm) containing standard food. Cap the vial and label with date of collection and genotype. House at 25°C, 65% relative humidity, 12‐hr/12‐hr light/dark cycle.
5b *For single‐housed (SH) experimental males*: Transfer 1 male fly each to individual housing vials (70 mm × 10 mm) containing standard food. Cap and label each vial. House under the same conditions as GH controls.
*IMPORTANT NOTE*: Flies should be divided into two groups, with some group housed and some singly housed. Single housing for 6 days substantially elevates male aggression relative to group housing. This is the standard pre‐assay condition used to produce robust, quantifiable aggression in Canton‐S males. Do not reduce isolation to <4 days, and do not extend isolation beyond 8 days before the assay.
6 House GH and SH vials in the same incubator to ensure identical temperature, humidity, and light cycle across conditions.
7 On the morning of the assay (Day 7), transfer vials from the incubator to the behavioral room at least 30 min before loading flies into arenas.

#### Scheduling assays by zeitgeber time

8 Perform aggression assays during ZT0‐ZT2.5 (the first 2.5 hr of the light phase). Record the clock time at the start of each assay session and verify it falls within the correct ZT window.
  Drosophila male aggression is strongly gated by circadian time. Assays performed outside this window will produce reduced behavioral output and increased inter‐dyad variability.
9 Perform courtship assays during ZT0‐ZT3 or ZT9‐ZT12. Use the same ZT window consistently within an experiment. Do not mix morning and evening sessions with the same dataset.

#### Preparing females for courtship assays (courtship experiments only)

Two methods of preparing female flies for experimentation are described below. Select the appropriate method based on the experimental design: mated females are used for standard courtship assays, whereas decapitated virgin females are used when assessing male courtship behavior independent of female behavioral feedback.

#### Option A: Mated females

10a On the same day as male collection (Day 1), collect newly eclosed virgin female flies. Anesthetize briefly on a CO_2_ pad and transfer females to group‐housing vials (∼20 females per vial).
11a To confirm virginity: Virgin females eclose with a whitish, expanded abdomen and meconium (dark material visible through the abdominal wall). Collect females within 6 hr of eclosion to ensure virginity.
12a Add 10 Canton‐S males to each female housing vial (∼20 females per vial) on the day of female collection to allow mating. House mixed vials at 25°C for 4‐6 days.
13a On Day 6 after collection (24 hr before the assay), transfer individual mated females from GH vials to individual holding vials containing standard food. On the morning of the assay (Day 7), load females directly into the arena by mouth aspirator without CO_2_ anesthesia (see Basic Protocol 2, step 11).

#### Option B: Decapitated virgin females

10b Collect virgin female flies 2‐4 days before the planned assay. House females in small groups without males at 25°C.
11b On the morning of the assay, confirm that females are 2‐4 days old. Anesthetize females on a CO_2_ pad. Under a stereomicroscope, hold the female body using a paintbrush to prevent movement and use fine microdissecting scissors to remove the head of each female with a single clean snip at the neck. Proceed immediately to arena loading. Decapitated females remain behaviorally active for ∼1 hr and must be loaded within this window. During loading, confirm that each decapitated female is mobile and responsive and occasionally grooms herself. Discard females that show no movement after decapitation.
*IMPORTANT NOTE*: Decapitation must be performed immediately before the assay, not in advance. Decapitate females in batches and load them into arenas in sequence. For experimental comparisons, each dyad must use a female that has not been used in any other dyad in the same experiment, as the dyad is the independent unit of replication. Reuse of a decapitated female across multiple arenas is permitted only when generating classifier training or validation data, where the goal is diverse annotated behavioral bouts rather than statistically independent replicates.

#### Time considerations

Collection and housing setup on Day 1 takes ∼1‐2 hr. No active work is required on Days 2‐5. On the morning of the assay (Day 7), allow 30 min for vial transfer and room acclimatization before loading. Preparation of mated females requires 5‐10 min, and preparation of decapitated virgin females requires <30 sec per female.

## Support Protocol 2 PREPARATION OF AGGRESSION FOOD AND ARENA COATING

This protocol describes the preparation of apple‐juice agar food plates for DANCE assays and the coating of blister‐pack arenas with Sigmacote. Both procedures must be completed on the day before behavioral recordings, as food plates require 8‐12 hr of drying and Sigmacote‐coated arenas require overnight drying. See recipe in Reagents and Solutions section for the full apple‐juice agar formulation.

### Materials

Apple juice, commercial (no added sugars or preservatives)
Agar (HiMedia, cat. no. GRM026)
Sucrose (HiMedia, cat. no. GRM601)
Sigmacote (Sigma‐Aldrich, cat. no. SL2)

Weighing balance
Measuring cylinder
500‐ml heat‐resistant beakers
Microwave oven or hot plate
Steel spatula
2‐mm acrylic base plate or food tray
Cheesecloth
Cling film (plastic wrap)
Razor blade
Refrigerator, 4°C
Cotton swabs (Solimo or equivalent)
Blister‐pack arenas
Fume hood

#### Part A: Food preparation

1 Measure 300 ml of commercial apple juice into a 500‐ml heat‐resistant beaker. Weigh out 9 g agar and add to the apple juice. Stir briefly to distribute the agar.
2 Heat the mixture in a microwave oven or on a hot plate, stirring intermittently with a steel spatula, until the agar is fully dissolved and the mixture reaches a rolling boil. Monitor closely to prevent boiling over.
  Agar dissolves slowly and requires sustained heating. Continue until no visible agar granules remain. Incomplete dissolution produces uneven food texture.
3 In a separate container, measure 100 ml apple juice and weigh out 10 g sucrose. Stir until completely dissolved. Heat for ∼2 min if dissolution is slow.
  Final composition: 2.25% (w/v) agar in commercial apple juice with 2.5% (w/v) sucrose (total volume: 400 ml).
4 Allow both solutions to cool briefly (2‐3 min) until no longer boiling. Pour the sucrose solution into the hot agar mixture. Stir thoroughly to achieve a uniform consistency.
*IMPORTANT NOTE*: Do not allow the combined mixture to cool below ∼50°C before pouring. If the agar sets prematurely, reheat gently once only. Repeated reheating reduces food quality.
5 Place acrylic food plates on a level surface. Pour the combined apple juice/agar mixture in a uniform layer across the food plate surface, aiming for a consistent thickness of ∼2‐3 mm.
6 Immediately remove any trapped air bubbles from the surface by drawing a steel spatula gently across the surface in one smooth motion.
*CAUTION*: Air bubbles create uneven surface texture that interferes with fly movement and affects behavioral output. Remove all bubbles before the agar begins to set.
7 Allow the food to solidify at room temperature for 5‐10 min, and then cover the surface loosely with cheesecloth.
8 Allow the covered food plate to dry at 25°C and 55%‐60% relative humidity for 8‐12 hr (overnight). At ambient humidity above 60% relative humidity, extend drying time to up to 16 hr. The surface is ready when it appears matte and nonreflective under direct light. A shiny surface indicates excess moisture; discard and re‐prepare.
9 After drying, carefully remove the food plate from the tray using a razor blade. Cut excess food from the edges to produce a clean, uniform surface.
10 Store prepared food plates at 4°C covered with cling film for up to 3 days. Allow refrigerated plates to equilibrate to room temperature for at least 30 min before use. Do not use plates where condensation has formed on the surface.

#### Time considerations

Active preparation time: ∼25 min. Overnight drying: 8‐12 hr.

#### Part B: Coating arenas with Sigmacote

*CAUTION*: Sigmacote (SL2) contains chlorotrimethylsilane in a heptane/xylene carrier. It is flammable and is a respiratory irritant. All coating steps must be performed in a fume hood. Keep away from open flames. Consult the Safety Data Sheet before use.

11 Bring Sigmacote and cotton swabs to a fume hood. Do not perform coating outside a fume hood.
12 Before use, gently squeeze the cotton tip of each swab to loosen the fibers. Compact cotton tips do not absorb adequate Sigmacote and will produce uneven coating.
13 Dip the loosened cotton tip into the Sigmacote bottle. Remove the swab and allow excess to drain briefly.
14 Apply Sigmacote to the inner walls and ceiling of each blister‐pack arena well using the saturated cotton swab. Coat in a circular motion to ensure complete coverage of all inner surfaces including the ceiling and upper walls.
15 Verify coverage by holding each coated arena up to a light source and rotating it. Uncoated patches appear as areas with different optical texture.
16 Allow coated arenas to air‐dry overnight at room temperature (minimum 8 hr). Do not use arenas before they are fully dry.
  Sigmacote older than 12 months may have reduced coating efficacy. If wall‐climbing is observed despite fresh coating, replace the Sigmacote stock.

#### Time considerations

Coating 8‐12 arenas takes ∼10‐15 min. Overnight drying (8‐12 hr) is required before use.

## Support Protocol 3 STANDARDIZED VIDEO ACQUISITION FOR DANCE RECORDINGS

This protocol describes the setup and configuration of the video recording system for DANCE behavioral experiments. Consistent recording parameters are required for reliable downstream tracking by FlyTracker. Complete this setup before loading flies into arenas (Basic Protocols 1 and 2).

### Materials

Electronic tablet or smartphone for backlight (compatible models tested: iPad Air, 5th generation; iPhone 13)
White screen light application (available in App Store and Google Play)
Transparent acrylic sheet
4‐mm acrylic spacers (4)
Assembled DANCE arena (see Basic Protocol 1 or 2)
Android smartphone camera (compatible models tested: Huawei Y9 2019; OnePlus Nord CE 2 Lite 5G, model CPH2381; Redmi Note 11 Pro+ 5G, model 221116SI) or Apple iPhone (compatible; before recording, navigate to Settings, then Camera, then Formats, and select Most Compatible; this records video in H.264 format as an .mp4 file, which is required for compatibility with the FlyTracker pipeline)
Camera mount or tripod (for fixed, vertical positioning)

1 Place the electronic tablet or smartphone on a flat, stable surface. Launch the white screen light application and set the display to a full‐screen white image. Adjust screen brightness to maximum or until the arena background appears uniformly bright when viewed from above.
2 Place the transparent acrylic sheet on top of the backlight device, supported by four 4‐mm spacers positioned at the corners. This creates a 4‐mm air gap between the screen and the acrylic sheet.
*IMPORTANT NOTE*: The 4‐mm air gap is not optional. Screen surfaces generate heat during extended use, and direct contact between the screen and the arena base raises arena temperature and suppresses behavioral output. Verify that the spacers are in place before placing arenas.
3 Place the assembled DANCE arena on top of the acrylic sheet, centered over the backlight display.
4 Mount the smartphone camera directly above the arena on a tripod or fixed stand. Position the camera so that the lens axis is perpendicular to the arena surface (pointing straight down). Adjust height so that all arenas are visible within the camera frame.
5 Disable autofocus before recording. For Android smartphones, navigate to Pro or Manual mode in the camera application, and then tap and hold on the arena surface in the viewfinder to lock focus. Confirm that the focus lock indicator appears and does not change when flies move. For iPhone, tap and hold on the arena surface in the viewfinder until the AE/AF Lock banner appears. Autofocus is now locked and will remain so for the duration of the recording.
*IMPORTANT NOTE*: Autofocus must be disabled. Changes in focus during recording produce video artifacts that interfere with FlyTracker background subtraction and will corrupt tracking for all frames after the focus shift.
6 Set the following recording parameters in the camera application:
  • Resolution: 1080p (1920 × 1080 pixels);
  • Frame rate: 30 fps;
  • Format: H.264 (.mp4);
  • White balance: set to a fixed value (Daylight or equivalent; do not use Auto white balance);
  • ISO: Set to the lowest available value that produces a well‐lit arena image (typically ISO 100 to 200).
  Specific menu paths for these settings vary across Android smartphone models. Consult the manufacturer's manual for the recording device in use.
7 Before loading flies, record a 10‐sec test clip of the empty arena. Review the clip and confirm that (a) the arena is fully in frame; (b) the food surface is visible through the well ceiling; (c) the image is in focus across all wells; and (d) there is no flickering due to backlight refresh rate.
  If flickering is visible in the test clip, adjust screen brightness or reduce the screen refresh rate to 60 Hz if the device permits.
8 Place the recording smartphone in airplane mode before beginning each recording session, to prevent incoming calls, notifications, or background processes from interrupting the recording. On the backlight device, disable auto‐lock or screen timeout to prevent the display from dimming during the session.
9 Record for 20 min for aggression assays and 15 min for courtship assays. Begin timing from the end of the acclimatization period (Basic Protocol 1, step 13, or Basic Protocol 2, step 13).

#### Time considerations

Initial setup of the backlight, acrylic spacer, and camera mount takes ∼10 min per session. Parameter configuration and test recording take 5 min. Recording takes 20 min (aggression) or 15 min (courtship). Total setup and recording time: 35‐40 min.

## Basic Protocol 2 CONSTRUCTION AND USE OF DANCE HARDWARE FOR RECORDING COURTSHIP BEHAVIORS IN *DROSOPHILA*

This protocol describes assembly of the DANCE hardware for courtship recording. The courtship arena uses the same blister‐pack and acrylic base‐plate components as the aggression setup (Basic Protocol 1), with the addition of thin slits cut into the top of each well to accommodate a separator comb made from repurposed X‐ray film. The separator divides each well into two compartments, allowing male and female flies to be loaded separately before the courtship session begins. Complete Support Protocol 1 (fly preparation) in advance and Support Protocol 3 (video acquisition setup) before loading flies. A video demonstration of DANCE courtship hardware assembly and recording is available as Figure 6—video 4 in Yadav et al. (2025; https://elifesciences.org/articles/105465/figures#fig6video4), and viewing this before beginning assembly is recommended for first‐time users.

### Materials

Male flies, some group housed and some singly housed (Support Protocol 1)
Female flies, mated or decapitated virgin (Support Protocol 1, option A or B)
Transparent medicine blister packs (standard courtship arena: 11 mm diameter × 4.5 mm height; alternatives: 13 mm × 5.5 mm and 17 mm × 4 mm)
Sharp razor blade
Repurposed X‐ray film (raw material for creating separator comb)
Scissors
White paper
Transparent tape
2‐mm‐thickness acrylic base plate with two loading holes per well (2‐mm diameter) positioned on either side of the well midpoint
1‐mm‐thickness acrylic side spacers
Paper tape
Mouth aspirator
Smartphone camera (Android or iPhone)
Electronic tablet or smartphone for backlight
Transparent acrylic sheet with 4‐mm spacers

1 Select transparent medicine blister packs with the appropriate well dimensions. The standard courtship arena measures 11 mm diameter × 4.5 mm height. Alternative sizes of 13 mm × 5.5 mm or 17 mm × 4 mm can be substituted, though larger arenas may show modestly reduced encounter rates (Yadav et al., 2025).
2 Select blister strips containing multiple wells in a row (5 wells per strip is standard). Using a sharp razor blade, cut a single thin slit through the top surface of each well, running across the well diameter. The slit should be narrow enough to hold the separator comb firmly while allowing the comb to be lifted cleanly at the start of recording.
*CAUTION*: Cut with controlled, gentle pressure. Excessive force will widen the slit or crack the blister material. A slit that is too wide will not hold the separator comb in position during fly loading.
3 Cut separator combs from a sheet of repurposed X‐ray film. Before cutting, draw the comb shape on a sheet of white paper: each tooth should be as wide as the inner diameter of the circular arena well (11 mm for the standard courtship arena) and tall enough to reach the loading base plate when inserted through the slit. The number of teeth should match the number of wells in the blister‐pack strip. Use this paper drawing as a stencil by placing it on top of the X‐ray film and cutting along the outline with scissors. Each tooth must span the full inner diameter of the well so that neither fly can pass into the other compartment before the comb is lifted. Store the paper stencil for reuse across sessions to ensure consistent tooth width.
4 After cutting, apply a strip of transparent tape across the top bar of the comb to create a gripping surface. The tape should be applied to the top bar only and must not extend onto the teeth, which need to sit flush against the loading base plate when the comb is inserted.
  The transparent tape handle makes it easier to grip and lift the comb cleanly at the start of recording without disturbing the flies or widening the slits. Lift the comb by holding the taped section between two fingers and pulling straight upward in a single smooth motion.
  Courtship arenas modified with separator slits are reusable for ∼10‐15 sessions. When slits begin to widen and the separator comb no longer holds position, replace the blister‐pack strip.
5 Insert the separator comb through the slits from above, with one tooth entering each well. The comb tooth should divide each well into two equal compartments, one for the male and one for the female.
6 Verify that each comb tooth reaches the loading base plate at the bottom of the well. A tooth that does not reach the bottom will not effectively separate the flies after loading.
7 Align the acrylic base plate (with two loading holes per well) under the blister‐pack strip so that each loading hole falls under one compartment of the corresponding well. Join the blister‐pack strip and base plate with paper tape along the long edges, using 1‐mm acrylic side spacers to keep the assembly aligned and stable.
8 Seal both short ends of the assembly with paper tape to eliminate any gaps that could allow fly escape.
9 Place the assembled arena on a flat surface for fly loading.
10 Using a mouth aspirator, load one male fly into the male‐side loading hole of each well. Confirm that the male has entered the well before sealing the loading hole with paper tape. Designate one side of every arena consistently as the male side and label the assembly before loading.
*IMPORTANT NOTE*: Do not anesthetize males before loading—transfer males directly from their housing vials into the arena by mouth aspirator, without CO_2_ exposure. Anesthesia on the day of the assay will suppress courtship and render the recording unusable.
  Some males should have been individually housed (experimental group) and some group housed (control group) for 6 days before this step (see Support Protocol 1, steps 5a and 5b). Do not load flies that appear lethargic or damaged.
11 Using a separate, clean aspirator tip, load one female fly into the female‐side loading hole of each well. If using mated females, use females prepared in advance (Support Protocol 1, option A: steps 10a‐13a). If using decapitated virgin females, load within 1 hr of decapitation (Support Protocol 1, option B: steps 10b and 11b). Seal the female‐side loading hole with transparent tape.
12 Place the loaded arena on the backlight setup, using a transparent acrylic sheet with 4‐mm spacers between the screen and the arena base.
13 Follow Basic Protocol 1, step 13, and allow flies to acclimatize in their respective compartments for 5 min with the separator comb in place. Keep the backlight on during acclimatization.
14 Position the smartphone camera above the arena in a fixed, vertical orientation on a tripod or mount. Configure recording parameters as described in Support Protocol 3. Record for 15 min.
15 After the 5‐min acclimatization period, gently lift the separator comb straight upward through the slits. Begin video recording immediately after comb removal. This moment defines the start of the courtship observation window.
*IMPORTANT NOTE*: Lift the separator comb vertically. Lateral movement widens the slits and will require the arena to be discarded. If the comb is difficult to remove, do not force it. Discard the arena and use a fresh one.

#### Time considerations

Separator combs and blister‐pack slits can be prepared in advance of the experimental session and stored for reuse across sessions. Assembly and sealing of one 5‐well strip takes ∼3 min. Loading males and females takes ∼3 min per strip. Acclimatization takes 5 min. Recording takes 15 min. Total per session for a single strip: ∼25 min. With practice, loading time decreases substantially from the estimates above. For higher throughput, multiple strips can be placed under separate smartphone cameras simultaneously, with two to four cameras running in parallel in a single session.

#### Notes

• Modified blister‐pack arenas are reusable for ∼10‐15 courtship sessions before slits widen.
• When slits widen and the separator comb no longer holds position, discard the strip and prepare a new one.
• Ensure that the separator comb fits snugly in all wells before loading flies.

## Basic Protocol 3 AUTOMATED QUANTIFICATION OF AGGRESSION AND COURTSHIP BEHAVIORS

This protocol describes the complete computational workflow for quantifying *Drosophila* aggression and courtship behaviors using the DANCE analysis pipeline. The pipeline proceeds in four stages: (1) video organization and chamber segmentation; (2) fly tracking using FlyTracker (Support Protocol 4); (3) behavioral classification using pretrained JAABA classifiers (Support Protocol 5); and (4) quantification of behavioral metrics using DANCE analysis scripts. The output is a set of .csv files containing per‐dyad behavioral indices suitable for statistical analysis. All DANCE analysis scripts are available at https://github.com/agrawallab/DANCE. Complete Support Protocols 2 and 3 before beginning.

### Materials

Recorded aggression videos (.mp4, H.264, 1080p, 30 fps, 20‐min duration) or courtship videos (.mp4, H.264, 1080p, 30 fps, 15‐min duration); see Support Protocol 3
Computer running Windows, macOS, or Linux
Python 3.8 or later (https://www.python.org); install dependencies using: pip install pandas numpy openpyxl matplotlib
Visual Studio Code (recommended Python IDE; https://code.visualstudio.com) or equivalent
MATLAB (R2020b or later; The MathWorks)
FlyTracker (v1.2.2; https://github.com/kristinbranson/FlyTracker)
JAABA (latest release from https://github.com/kristinbranson/JAABA; do not use the SourceForge distribution, which lacks functions required for courtship classifier feature computation)
DANCE classifier files (.jab format; download from https://drive.google.com/drive/folders/1N75OinwMuYrkIT3qclAJThCvfOZMuotj)
DANCE analysis scripts (download from https://github.com/agrawallab/DANCE):
Auto_Cropper.py (video segmentation)
Lunge_Analyzer_indiC.py (aggression quantification)
courtship_bout_num_extractor.py (courtship quantification, segmented videos)
courtship_bout_num_extractor_unsegmented.py (courtship quantification, unsegmented videos)
JAABA_path_extractor.py (compiles experiment folder paths for batch classification in JAABA)
organize.py (creates one folder per recording)

#### Video organization

1 Create a master project directory. Inside it, create a subfolder named with the recording date in DDMMYYYY format (e.g., 07052026). Place all raw video files (.mp4) from a single recording session into this date folder.
2 Verify that all videos meet the following specifications before proceeding: .mp4 format, H.264 encoding, 1080p resolution, 30‐fps frame rate. Open one video in a media player and confirm that fly bodies are visible against the backlit background with high contrast. If contrast is insufficient, re‐record using corrected lighting settings (Support Protocol 3, steps 1‐4).
3 Copy the organize.py script into the directory containing the raw video files from a single recording session. Open organize.py in Visual Studio Code and execute it by clicking the Run (Play) button. The script automatically creates a separate folder for each recording and organizes the corresponding files into their respective directories, thereby establishing a standardized folder structure for downstream processing. Successful completion of the process is indicated by the message “Organization complete.” displayed in the terminal.

#### Video segmentation using auto cropping

4 Open Auto_Cropper.py in Visual Studio Code by double‐clicking the file. Click the Play button to run the script.
5 The terminal will prompt: “What is the path of the master video folder?” Copy and paste the full path of the date folder and press Enter.
6 The terminal will prompt again: “How many chambers are there per video?” Enter the number of chambers in the arena and press Enter.
7 The script segments each video and writes all cropped chamber videos into a single output folder. This structure allows one calibration file to be applied to every video from the session during tracking (see Support Protocol 4).
8 Before proceeding, open the preview image saved alongside the output and confirm that every chamber has been correctly identified. If any chamber is missed or a spurious circle appears, adjust the circle detection parameters (minRadius, maxRadius, and param2) in Auto_Cropper.py to match the pixel size of the chambers in your recordings, and rerun segmentation. These parameters are specific to the camera and arena configuration in use and are not universal defaults.
9 When segmentation is complete, the terminal will display the message: “Cropped videos for X circles have been saved.” This confirms successful segmentation.
  Ensure that the master video folder contains the necessary input video files before running the script. The output folder will be structured as required for FlyTracker. If any error occurs, verify that the correct folder path and number of chambers were provided.

#### Fly tracking and behavioral classification

10 Track all segmented chamber videos using FlyTracker. Follow Support Protocol 4 in full. Confirm that each segmented chamber folder contains a [videoname]_JAABA/ subfolder with trx.mat and a perframe folder before proceeding.
11 Generate behavioral score files using the pretrained DANCE classifiers. Follow Support Protocol 5 in full. For aggression experiments, load the Lunge_oneframe_classifer.jab classifier only. For courtship experiments, load all five courtship classifiers. Confirm that score files (e.g., scores_Lunge.mat) are present in each _JAABA/ folder before proceeding.

#### Aggression quantification (lunge analysis)

Perform steps 12a‐14a for aggression experiments only.

12a Open Lunge_Analyzer_indiC.py in Visual Studio Code. Click the Play button to run the script.
13a The terminal will prompt: “What is the complete path of the parent folder containing all videos and JAABA folders?” Enter the full path of the date folder and press Enter.
14a The script will process all lunge score files and generate a compiled .csv output file in the parent directory. Because the lunge classifier is a single‐frame classifier, each detected lunge is a discrete event rather than a bout with a measurable duration. The output contains one row per chamber, reporting the lunge frame numbers for each fly and the total lunge count for the dyad across the 20‐min session. Verify that the number of rows matches the number of dyads recorded.

#### Courtship quantification

Perform steps 12b and 13b for courtship experiments only.

12b Open courtship_bout_num_extractor.py in Visual Studio Code (or, if videos were not segmented into individual chamber folders, use courtship_bout_num_extractor_unsegmented.py). Click the Play button to run the script.
13b The terminal will prompt: “What is the complete path of the parent folder containing both video and JAABA folders?” Enter the full path of the date folder and press Enter. The script will rename and organize score files into a centralized score_files/ folder, and compile behavioral data from all five courtship classifiers into per‐classifier .csv files. Proceed to step 15.
  Male identity assignment in the courtship output is based on the assumption that the fly with the higher wing extension count is the male. If the experimental design uses male‐male pairs, verify identity assignments manually.

#### Output verification

15 Confirm that the following output files are present. Aggression: one compiled .csv file with lunge counts per dyad. Courtship: one compiled .csv file per classifier (wing extension, circling, following, attempted copulation, copulation), each containing behavioral indices per dyad. If the genotype or manipulation under study may reduce wing extension, also verify identity assignments manually on a subset of videos before analyzing a new experiment type.
16 Each courtship output .csv contains one row per recorded dyad and three columns: Fly 1 output, Fly 2 output, and assigned male identity. Male identity is derived from the wing extension assumption described above and is the value recommended for downstream analyses. The aggression output is structured differently (see step 14a).

#### Time considerations

Video organization and directory setup: ∼5 min. Chamber segmentation: ∼2‐5 min for eight videos. FlyTracker tracking for eight dyads: ∼10‐15 min (see Support Protocol 4). JAABA classification for eight dyads with a single classifier: ∼5‐10 min; with all five courtship classifiers: ∼20‐30 min (see Support Protocol 5). Running Lunge_Analyzer_indiC.py or courtship_bout_num_extractor.py: <1 min. Total time from organized raw videos to analyzed .csv output: ∼25‐60 min depending on experiment type.

## Support Protocol 4 ANIMAL TRACKING USING CALTECH FlyTracker

This protocol describes how to track fly movements in segmented DANCE videos using FlyTracker, a freely available MATLAB‐based software that tracks the pose and maintains the individual identity of each fly across video frames (Eyjolfsdottir et al., 2014). Complete this protocol after video segmentation (Basic Protocol 3, steps 4‐9). The output is a trx.mat tracking file and a per‐frame features folder inside a JAABA‐compatible directory, which are required inputs for Support Protocol 5. Representative FlyTracker calibration, tracking, and identity swap correction outputs are shown in Figure 2. Tracking one batch of eight videos takes ∼25‐35 min.

**Figure 2:**
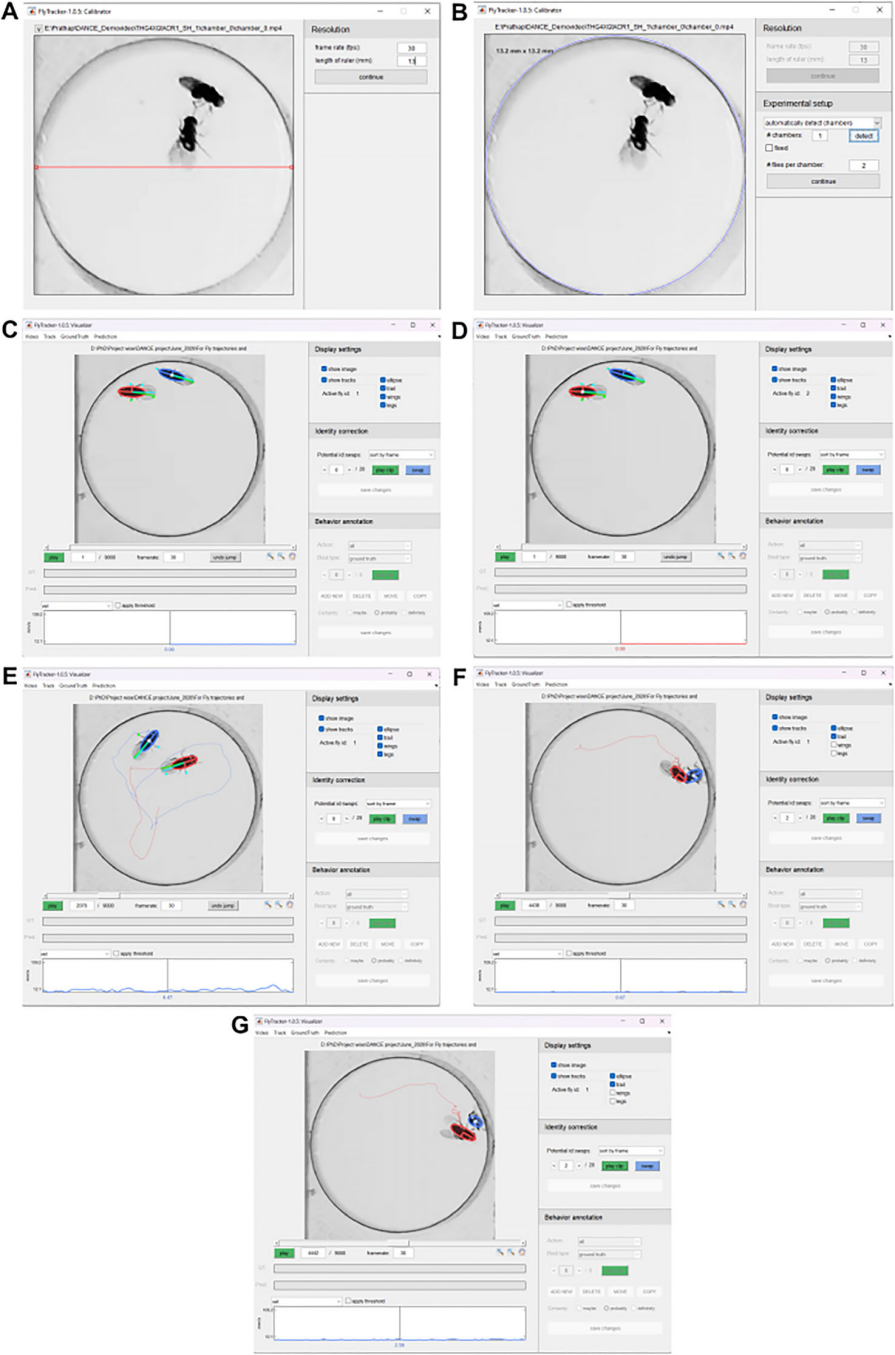
Representative FlyTracker workflow. (**A**) Screenshot of FlyTracker calibration window showing correctly placed red boundary markers aligned to arena edges to define the arena diameter. (**B**) FlyTracker automatically identifies the arena boundary as a blue circle. (**C and D**) Visualization of tracked flies in the FlyTracker Viewer. Individual flies are assigned unique identities and displayed as color‐coded ellipses, each marked with a white star (Fly 1: blue; Fly 2: red). (**E**) Fly trajectories generated from tracking data. Colored traces represent the movement paths of individual flies over time. (**F**) Example of an identity‐swap error detected during tracking. During close‐proximity interactions, the identities of the two flies are incorrectly labeled, resulting in erroneous trajectory assignments that require manual correction. (**G**) Corrected tracking output following identity correction in the FlyTracker Viewer. Fly identities and trajectories are restored, ensuring accurate tracking data for downstream behavioral analysis and JAABA classification. Figure adapted from Yadav et al. (2025).

### Materials

MATLAB (R2020b or later; The MathWorks; https://www.mathworks.com)
FlyTracker (v1.2.2; https://github.com/kristinbranson/FlyTracker; see step 1)
JAABA (latest release from https://github.com/kristinbranson/JAABA; must be installed and added to the MATLAB path before tracking so that FlyTracker can generate JAABA‐compatible output; see step 2)
Segmented video folders (output of Auto_Cropper.py; see Basic Protocol 3)

*NOTE*: Both FlyTracker and JAABA must be added to the MATLAB path before beginning. In MATLAB, navigate to Home, then Set Path, then Add with Subfolders. Select the FlyTracker root folder, click Save. Repeat for the JAABA root folder. This step is required only once per MATLAB installation.

#### Installation (first use only)

1 Download FlyTracker from https://github.com/kristinbranson/FlyTracker. Follow the installation instructions in the repository README. Confirm installation by typing tracker in the MATLAB command window. The FlyTracker GUI should open.
2 Download JAABA from https://github.com/kristinbranson/JAABA. Do not use the SourceForge distribution, which lacks functions required for courtship classifier feature computation. Follow installation instructions. Confirm by typing JAABAPlot in the MATLAB command window.
3 Add both FlyTracker and JAABA to the MATLAB path with subfolders as described in the Materials note above. Save the path.

#### Video loading

4 In the MATLAB command window, type tracker and press Enter. The FlyTracker GUI will open.
5 Click Video Folder. In the dialog box, navigate to and select the folder containing all cropped arena videos from the recording session, produced by Auto_Cropper.py (Basic Protocol 3). Click Select Folder.
  For batch processing, all cropped arena videos from the same recording session are stored in a single folder by Auto_Cropper.py. FlyTracker applies one calibration file to every video in that folder. This is valid provided that arena size, camera position, and recording conditions are identical across the session, which is the case for videos segmented from a single recording.

#### Calibration

6 Click Calibrate. A dialog box titled Loading Calibrator will appear, followed by a frame from the video with red boundary markers overlaid on the arena.
7 Click and drag the red markers to align precisely with the left and right inner edges of the circular arena boundary.
*IMPORTANT NOTE*: Inaccurate marker placement will cause FlyTracker to miscalculate the spatial scale of the video and will invalidate all downstream distance and velocity measurements. Align markers carefully, as this calibration will be applied to all videos in the folder.
8 In the right panel under Resolution, locate the Length of the ruler (mm) field. Enter 13 for the standard 13‐mm diameter aggression arena. Enter the appropriate diameter if using an alternative arena size (17 mm or 21 mm; see Basic Protocol 1, step 1), or 11 mm for the standard courtship arena and alternative arena size (13 mm or 17 mm; see Basic Protocol 2, step 1).
9 Click Continue. A progress dialog titled Computing background image will appear. MATLAB will display frame numbers in the command window as it processes the video. For a 20‐min video at 30 fps, 36,000 frames will be processed. Wait until the Background panel displays the Bg Mean, Bg Variance, and Sample Image.

#### Experimental setup

10 Under Experimental Setup, enter 1 in the # Chambers field for individual segmented chamber videos (typically 1 arena per video). For multichamber recordings, enter the correct number.
11 Click Detect. A progress dialog will appear, and detected arena boundaries will be displayed as blue circles. If arena detection fails or produces incorrect boundaries, click the dropdown menu under Automatically Detect Chambers and select Manually Set Chambers. Adjust the arena boundary by clicking and dragging.
12 Verify that the # Flies Per Chamber field shows 2. If the software detects a different number, manually set it to 2.
13 Click Continue.

#### Parameter tuning and tracking

14 Adjust the Foreground Threshold slider until the fly body, legs, and wings are consistently highlighted in the preview window. Start with the default value and adjust in increments of 5.
15 Adjust the Body Threshold slider until the body outline of each fly is cleanly segmented from wings and legs. The color indicators in the corner of the calibration window show which pixels are classified as body versus wing.
*IMPORTANT NOTE*: Over‐thresholding will merge the two fly bodies into a single detected object. Under‐thresholding will fragment one fly into multiple objects. If either condition persists despite adjustment, recheck video contrast (Support Protocol 3) before proceeding.
16 Click Finish. A dialog box titled Finalizing Calibration will appear.
17 Enter the number of CPU cores to use for parallel processing. Reserve two or three cores for operating system processes when selecting this value. Using all available cores risks freezing the computer. Click OK.
18 Ensure that the Save JAABA Folders checkbox is selected. Click TRACK. FlyTracker will automatically apply the calibration settings to all videos within the selected folder and process them in batch mode. Do not close MATLAB or interact with the GUI during tracking.
19 After tracking is successfully completed, navigate to the video folder and confirm that the following output files and folders are present in each video folder: [videoname]_JAABA/ (the JAABA‐compatible experiment directory); [videoname]_JAABA/trx.mat (the tracking data file); and [videoname]_JAABA/perframe/ (the per‐frame features folder containing .mat files for each computed feature).
  If the perframe folder is absent or empty, tracking did not complete successfully. Check the MATLAB command window for error messages. The most common cause is an incorrect number of flies per arena (step 12). Correct the value and rerun from step 10.
  Using a single calibration file for all the cropped arena videos substantially reduces analysis time while maintaining consistent tracking parameters across all arena videos from the session. This approach is recommended whenever arena geometry and recording conditions remain unchanged throughout the recording session.
20 Repeat steps 4‐19 for each folder in the dataset.
  Alternatively, if arena positions, lighting conditions, or recording geometry vary between videos, proceed to steps 21‐23 to calibrate each arena video independently.

#### One‐to‐one calibration for individual arena videos

21 The video segmentation script can be configured to save each cropped arena video into a separate folder.
22 Open FlyTracker and select the folder containing a single arena video. Perform calibration, chamber detection, parameter tuning, and tracking as described above.
23 Repeat the calibration procedure for every arena video in the dataset.
  This approach is substantially more time consuming than batch processing with a shared calibration file and should be used only when necessary. One‐to‐one calibration may improve tracking accuracy when experimental conditions vary between arenas or recording sessions.

#### Identity swap verification and correction

24 After tracking all videos, inspect each result for identity swaps. In the FlyTracker GUI, click Visualizer and load the tracking output folder.
25 Step through the video using the frame slider. Identity swaps appear as an abrupt crossing of the two fly trajectories. Note the frame numbers where swaps occur.
26 Use the built‐in correction tool to manually correct identity swaps at the swap frame. Save the corrected tracking data.
  A small number of identity swaps are expected during tracking, particularly when flies overlap or come into close contact. Potential identity swaps are automatically flagged by the FlyTracker Visualizer and should be manually inspected. Inspect every video as described in steps 24‐26, rather than only the flagged frames. Although a small number of identity swaps may occur, particularly during occlusions, the overall identity switch rate is typically low and does not substantially affect behavioral quantification.
27 Re‐save the corrected tracking data. The trx.mat file will be overwritten with the corrected version.

#### Time considerations

Installing FlyTracker and JAABA and adding them to the MATLAB path is a one‐time setup requiring ∼30 min. For a standard aggression experiment with eight dyads, tracking takes ∼10‐15 min total across all eight videos. Identity swap verification and correction for eight videos takes ∼15‐20 min. Total time for this protocol with a standard eight‐dyad dataset: ∼25‐35 min.

## Support Protocol 5 SCORE FILE GENERATION USING JAABA CLASSIFIERS

This protocol describes how to classify fly behaviors in tracked DANCE videos using JAABA with pretrained DANCE classifiers. Complete Support Protocol 4 (FlyTracker tracking) before beginning. The inputs are the JAABA‐compatible output folders produced by FlyTracker. The outputs are per‐frame behavioral score files (.mat format) for each of the six DANCE classifiers: lunge, wing extension, circling, following, attempted copulation, and copulation. Classification of one batch of 8 videos takes ∼5‐10 min.

### Materials

FlyTracker output folders (each containing a [videoname]_JAABA/ directory with trx.mat and perframe/ subfolder; see Support Protocol 4)
MATLAB (R2020b or later; The MathWorks)
JAABA (latest release from https://github.com/kristinbranson/JAABA; do not use the SourceForge distribution, which lacks functions required for courtship classifier feature computation)
DANCE classifier files (.jab format; download from https://drive.google.com/drive/folders/1N75OinwMuYrkIT3qclAJThCvfOZMuotj):
Lunge_oneframe_classifer.jab (aggressive lunge)
WingExt.jab (wing extension during courtship)
Circling.jab (circling behavior)
Following.jab (following behavior)
Attempted_Copulation.jab (attempted copulation)
Copulation.jab (copulation)

*NOTE*: JAABA and FlyTracker must both be on the MATLAB path before JAABA is launched. If MATLAB has been restarted since Support Protocol 4, re‐add both folders: Home, then Set Path, then Add with Subfolders. Select FlyTracker root, save; repeat for JAABA root.

#### Processing experiments in batch

To enable batch classification of multiple experiments in JAABA, the paths to all experiment folders must first be compiled into a text (.txt) file. This is automated using the JAABA_path_extractor.py script. Open JAABA_path_extractor.py in Visual Studio Code and execute it by clicking the Run (Play) button. When prompted, provide the path to the parent folder containing all tracked experiment folders from a single recording session (e.g., 07052026). The script automatically generates the paths of all JAABA experiment folders within the specified directory and generates a text file named JAABA_paths.txt in the parent folder. This file contains the paths to all experiment folders and serves as the input for subsequent batch classification in JAABA.

#### Launch JAABA

1 In the MATLAB command window, type JAABAPlot and press Enter. The JAABA main window will open.

#### Create a new project

2 In the JAABA window, under the Experiments section, click New. Enter a project name based on the recording date (e.g., 07052026). Click OK.
3 A color selection dialog box will appear. Select distinct, color‐blind‐friendly colors for each experiment label. Click OK.

#### Add tracked experiments

4 Under the Experiments section, click Batch. Navigate to the folder containing JAABA_paths.txt. Double‐click to open the .txt file.
5 Alternatively, under the Experiments section, click Add. Navigate to the folder containing a tracked video. Double‐click to open it, then double‐click on the [videoname]_JAABA subfolder to select it. Click Done.
*IMPORTANT NOTE*: You must select the _JAABA subfolder specifically, not the parent video folder. JAABA requires the directory that contains the trx.mat file and perframe/ folder. Selecting the wrong level will cause JAABA to fail to load experiment data.
  Manual addition of experiments using the Add option is considerably more time consuming and less efficient than batch processing, as each experiment folder must be selected individually. Therefore, batch processing is recommended whenever multiple experiments (large number of recordings) are to be analyzed.
6 Repeat step 5 for each additional tracked video. Verify that all experiments appear in the Experiments panel.

#### Load classifiers

7 Under the Classifiers section, click Add. Navigate to the folder where the DANCE .jab classifier files are stored.
8a *For aggression experiments*: Select Lunge_oneframe_classifer.jab and click Open.
8b *For courtship experiments*: Navigate to the directory containing the classifier files, select WingExt.jab, Circling.jab, Following.jab, Attempted_Copulation.jab, and Copulation.jab simultaneously (using multi‐select), and click Open. All selected classifiers will be loaded into JAABA in a single step.
  For a standard aggression recording, load only Lunge_oneframe_classifer.jab. Loading all six classifiers for an aggression recording will produce empty score files for the courtship behaviors and will extend processing time unnecessarily.

#### Run classification

9 Click Classify. JAABA will begin processing all loaded experiments against all loaded classifiers. Do not close MATLAB or interact with the JAABA window during classification.
10 Wait until the status display shows Ready for all experiments.
11 Verify that classification completed successfully. Navigate in the file system to the [videoname]_JAABA/ folder for one processed video. Confirm that score files are present: scores_Lunge.mat (aggression experiments); scores_WingExt.mat, scores_Circling.mat, scores_Following.mat, scores_Attempted_Copulation.mat, and scores_Copulation.mat (courtship experiments).
  If score files are absent after classification completes, the most common cause is a mismatch between the frame rate of the videos used to train the classifier and the frame rate of the new videos. Verify that all videos were recorded at 30 fps (Support Protocol 3, step 6).
12 Repeat the classification process (steps 2‐11) for any additional recording sessions not yet processed.

#### Post‐classification: organize score files for analysis

After classification is complete across all sessions, see Basic Protocol 3 for automated quantification of aggressive lunges (steps 12a‐14a) and courtship behaviors (steps 12b and 13b).

#### Time considerations

For a standard eight‐dyad aggression experiment (eight videos, lunge classifier only), classification takes ∼5‐10 min. For a courtship experiment (eight videos, five courtship classifiers), classification takes ∼20‐30 min.

## Alternate Protocol 1 TRAINING CUSTOM JAABA CLASSIFIERS

This protocol describes how to train a new JAABA classifier for a behavior not covered by the existing DANCE classifiers, or to retrain an existing classifier on videos recorded under experimental conditions that differ substantially from those used to generate the original training set, for example a different arena geometry, a different fly strain with an unusual body size, or a novel behavior of interest. Use this protocol when pretrained classifiers produce systematically low or inconsistent scores despite correct FlyTracker tracking and JAABA setup. Complete Support Protocol 4 on all videos intended for training and testing before beginning.

*NOTE*: Training a JAABA classifier is an iterative process requiring multiple rounds of annotation and retraining. Allow at least 2‐3 days for an initial classifier. Do not train a classifier on fewer than five independent videos.

### Materials

Tracked videos with FlyTracker output (_JAABA/ folders containing trx.mat and perframe/; see Support Protocol 4), at minimum 15 videos for training and testing combined, and 20‐30 is recommended
MATLAB (R2020b or later; The MathWorks)
JAABA (latest release from https://github.com/kristinbranson/JAABA; do not use the SourceForge distribution, which lacks functions required for courtship classifier feature computation)
Written behavioral definition for the target behavior (see step 1)

#### Preparing for annotation

1 Before beginning annotation, write a precise operational definition of the target behavior specifying: (a) the postural or kinematic features that characterize the behavior; (b) the minimum duration constituting a true bout in frames; and (c) criteria for distinguishing the behavior from closely related behaviors. All annotators must use the same written definition. Inconsistent annotation is the most common cause of poor classifier performance.
2 Divide tracked videos into two groups before any annotation begins: a training set (∼70% of videos) and a test set (remaining 30%). The test set must not be used during training. Do not open test videos in JAABA until the classifier is trained; they are annotated separately as ground truth in Alternate Protocol 2. Record the assignment of each video to train or test set before opening any video in JAABA.
*IMPORTANT NOTE*: If a test video is examined and annotated before training is complete, it is contaminated and cannot be used for unbiased validation. Discard contaminated test videos and designate new ones from the training pool.

#### Setting up a JAABA project

3 In the MATLAB command window, type StartJAABA and press Enter.
4 A dialog box named “JAABA Open” will appear. Under the JAABA Open window, click New. In the New dialog box, under Target Type, select eyrun_flies from the drop‐down menu. Enter a descriptive name for the behavior to be classified (e.g., wing_flick) in the Behavior Name field. Specify the Movie File Name (e.g., movie.mp4) and Tracking Data File Name (trx.mat). JAABA will automatically generate the corresponding Output Score File Name (e.g., scores_wing_flick.mat). Leave the Advanced parameters at their default settings, and click Done to create the new classifier project.
5 Under the Experiments section, click Add. Navigate to and select the _JAABA subfolder of the first training video. Make sure that the folder contains movie.mp4, the perframes folder, and the trx.mat file, and then click Done. Repeat for each remaining training video. Do not add any test set videos at this stage.

#### Annotating training bouts

6 The video and trajectory display will load in the JAABA window.
7 Once they are loaded, navigate through the video using the frame slider. When a behavioral bout matching the written definition from step 1 is identified, mark the corresponding frames by clicking on the “Behavior” label at the top right corner (red box). Mark frames that clearly do not contain the behavior by clicking on None (blue box).
*IMPORTANT NOTE*: JAABA requires both positive examples (labeled as Behavior) and negative examples (labeled as None) to train a classifier. Actively label clear non‐bout frames, especially those that superficially resemble the target behavior. If only positive bouts are labeled, JAABA treats all other frames as ambiguous, which reduces classifier specificity. Ensure that the Behavior and the None labels are roughly balanced to avoid prediction bias.
8 After annotating a few bouts and non‐bouts, the classifier can be trained by clicking on the “Train” button at the bottom right. The status bar will indicate that the training is running.
9 When you click on “Train” for the first time, it will ask you to select features for training. Once you click OK, a window for “Select Features” will pop up. Choose the per‐frame categories you want to include using All or None in the Select column. Set the Amount to normal, enter a positive integer for Window Size, and click Done to continue.

#### Iterative refinement

10 In the frame window, you will be able to see the predictions of the classifier based on the labeling you had done before “Train.” Darker colors indicate low confidence, while brighter colors indicate high confidence. However, some frames will not be labeled for prediction, for which you can click on the “Predict” at the bottom.
11 Review the predictions and identify false positives (frames labeled as behavior that are not genuine bouts) and label them as None in JAABA. Identify false negatives (genuine bouts that the classifier missed) and label them as Behavior.
12 Once you have finished correcting the labels, click Train again to retrain the classifier. You can compare the updated predictions with the previous results by selecting “Old” from the drop‐down menu at the bottom of the automatic predictions timeline.
13 The predictions can be made better, if needed, by using the Post‐processing option from the Classifier dropdown.
14 To quickly measure the classifier's accuracy and precision, go to the Classifier tab and select Cross‐validate. You will get a table showing all the errors made by the classifier based on the manual labels and the predictions.
15 Repeat steps 11‐14 until classifier performance on the training videos appears stable across iterations, meaning that three consecutive additions produce no improvement in precision and recall.
  The following minimum training data volumes from the DANCE eLife study provide a reference for the labeled frames required to produce well‐performing classifiers (Table 1).
  These values represent cumulative annotation volume across all training videos, pooled across many recordings and many dyads. They are not measurements of any single assay or behavioral bout.

**Table 1:** Training Data Volumes for JAABA Classifiers

Behavior	Manually labeled frames (pooled across all training videos)	Equivalent video duration at 30 fps
Lunge	1,449	∼48 s
Wing extension	99,947	∼3,332 s
Attempted copulation	39,513	∼1,317 s
Copulation	56,979	∼1,899 s
Circling	14,396	∼480 s
Following	25,787	∼860 s

Table 1 lists the training data volumes used for the DANCE classifiers in the eLife study as a reference. These are not established minimum requirements; the amount of labeled data needed depends on the behavior and the recording conditions.

#### Saving the classifier

16 When classifier performance on training videos is satisfactory, click File, then Save Classifier. Save as a .jab file with a descriptive filename that includes the behavior name and date (e.g., wing_flick_07052026.jab).
17 Proceed to Alternate Protocol 2 to validate the saved classifier on the held‐out test set before using it for experimental data.

#### Time considerations

Setting up the JAABA project and completing initial annotation of three to five training videos takes ∼5‐7 hr. Each subsequent refinement round takes 1‐2 hr. A full training run for a lunge‐type single‐frame classifier typically requires 4‐5 days. These estimates are approximate and depend on annotator experience and computing resources. Duration‐based behaviors may require 5‐7 days due to large training frame requirements.

## Alternate Protocol 2 GROUND‐TRUTH ANNOTATION AND CLASSIFIER VALIDATION

This protocol describes how to generate manual ground‐truth behavioral annotations on held‐out test videos and use them to evaluate the performance of a newly trained JAABA classifier (Alternate Protocol 1). This validation step must be completed before using any custom classifier for experimental data analysis. The same workflow can also be used to benchmark the pretrained DANCE classifiers on new hardware or recording conditions that differ from those used in the original validation study.

### Materials

Test videos with FlyTracker output (_JAABA/ folders; see Support Protocol 4); a minimum of 10 videos not used in training (15‐20 videos covering the full range of behavioral intensities recommended)
JAABA classifier .jab file (pretrained classifiers from https://drive.google.com/drive/folders/1N75OinwMuYrkIT3qclAJThCvfOZMuotj; custom classifiers from Alternate Protocol 1)
MATLAB (R2020b or later; The MathWorks)
JAABA (latest release from https://github.com/kristinbranson/JAABA)
Python 3.8 or later; pandas, numpy, openpyxl libraries installed
DANCE analysis scripts from https://github.com/agrawallab/DANCE:


extractLabels
individual_video_manual_labels
Sheetnames_maker
manual_JAABA_mateBook_to_workbook
Overlap_analyzer
behavior_index_matebook_table
Classification_metrices_table

#### Manual annotation of test videos

1 In the MATLAB command window, type StartJAABA and press Enter.
2 A dialog box named “JAABA Open” will appear. Under the JAABA Open window, click Open in Ground Truthing Mode, and it will ask for the .jab file (developed classifier); click on the relevant classifier .jab file. Once loaded, go to File and click on the Change Experiment list and add all the test set videos.
*IMPORTANT NOTE*: Manual annotation must be completed before the classifier is run on any test video. Examining classifier predictions before completing manual annotation introduces observer bias and invalidates the validation. Do not run the classifier on, or view its predictions for, any test video until manual annotation of all test videos is finished (step 5). Predictions are generated in step 6.
3 Select the first test video from the Experiments panel. Navigate through the video using the frame slider.
4 Identify behavioral bouts matching the written behavioral definition (see Alternate Protocol 1, step 1). For each genuine bout, select the corresponding frames and click on Behavior. For frames that clearly do not contain the behavior, click on None. Annotate each test video completely before moving to the next.
  If two annotators are available, annotate at least 20% of test videos independently and compare results using a non‐parametric test before proceeding. If inter‐annotator agreement is low (Cohen's kappa <0.6), revisit the behavioral definition in Alternate Protocol 1, step 1.
5 Repeat step 4 for all test videos. Save the JAABA project after completing each video.

#### Running the classifier on test videos

6 After annotating all test videos manually, load the classifier .jab file: under the Classifiers section, click Add and select the .jab file. Click Classify. Wait until all experiments show Ready status.
7 Verify that score files (scores_[behaviorname].mat) are present in the _JAABA/ folder for each test video before proceeding.

#### Extracting and comparing annotations

8 Run the extractLabels script on the JAABA project file containing manual ground‐truth annotations. This script reads the .jab file and exports manually labeled bout boundaries to a test.csv file. Input: test.jab; output: test.csv.

#### For duration‐based classifiers

9 Run the individual_video_manual_labels script on the test.csv output from step 8. This script calculates per‐video bout counts and produces individual bout files for each video and a summary file video_path_counts.csv listing total bout counts per video.
10 Run the Sheetnames_maker script, providing the list of video names from video_path_counts.csv as input. This script generates an empty Excel workbook, test_Workbook.xlsx, with one sheet per test video and a standard header row. Output: test_Workbook.xlsx.
11 Organize manual output and JAABA classifier output into separate subfolders. Run manual_JAABA_mateBook_to_workbook to compile all outputs into the test_Workbook.xlsx. Input: folder containing manual output, JAABA output, and the test_Workbook.xlsx file; output: populated test_Workbook.xlsx.

#### Calculating performance metrics

12 Run the Classification_metrices_table script on the completed test_Workbook.xlsx. This script compares predicted and ground‐truth bouts using the following criteria: a predicted bout is scored as a true positive (TP) if it overlaps with a ground‐truth bout by at least one frame (∼33 ms at 30 fps). Predicted bouts with no overlap are scored as false positives (FP). Ground‐truth bouts with no overlapping prediction are scored as false negatives (FN). Output: precision, recall, and *F*_1_ score for each test video, and the same metrics calculated across all test videos combined. Precision is calculated as TP divided by (TP plus FP). Recall is calculated as TP divided by (TP plus FN). *F*_1_ score is calculated as 2 multiplied by (Precision multiplied by Recall), divided by (Precision plus Recall).
13 Additionally, run the behavior_index_matebook_table script on the completed test_Workbook.xlsx. This script calculates a per‐video behavior index across different platforms, for a direct comparison between behavior indices derived from ground‐truth annotations and those predicted by the JAABA classifier. The behavioral index for courtship classifiers is calculated as the proportion of total frames classified as the behavior: behavioral index equals total frames classified as behavior divided by total frames in the recording.
  To calculate performance metrics for a single‐frame classifier such as the lunge classifier, use the Overlap_analyzer script from the GitHub repository. The input file is an Excel sheet containing the manually annotated frames and the JAABA‐predicted frames; the output is the per‐video TP, FP, and FN counts.

#### Interpreting results and acceptance criteria

14 Evaluate classifier performance against the reference values from the DANCE eLife validation study (Table 2).

**Table 2:** Classifier Performance on the Held‐out Test Set in the eLife Validation Study

Classifier	Precision	Recall	*F*_1_ score
Lunge	78.7%	73.1%	75.8%
Wing extension	92.2%	98.1%	95.1%
Circling	98.0%	92.1%	95.0%
Following	91.2%	91.1%	91.1%
Attempted copulation	82.6%	89.2%	85.8%
Copulation*^a^*	100%	100%	100%

Copulation is operationally defined as mounting lasting ≥45 sec, making it a temporally discrete and visually unambiguous event. This behavioral clarity accounts for the near‐perfect bout‐level classification performance. Frame‐level performance metrics, which are marginally lower across all classifiers including copulation (precision 98.17%, recall 95.55%, *F*_1_ 96.85%), are reported in Figure 5—figure supplement 3 of Yadav et al. (2025).

15 For a newly trained custom classifier, an *F*_1_ score of ≥0.70 on the held‐out test set is the minimum acceptable performance before using the classifier for experimental data. An *F*_1_ score <0.70 indicates that the classifier is undertrained or trained on insufficiently diverse data, or the behavioral definition requires revision. The 0.70 threshold is a general guide; the acceptable score depends on the experimental question, so also check classifier predictions against the video to confirm that the interactions relevant to the question have been captured.
16 If the *F*_1_ score is below the acceptance threshold, return to Alternate Protocol 1, step 11, and add further training examples. A high FP rate indicates that the classifier is too liberal and requires more negative examples. A high FN rate indicates that the classifier is missing true bouts and requires more positive examples from the behavioral intensity range in which misses occur.
17 Once the classifier meets the acceptance threshold, record the test set performance metrics in the experimental lab notebook alongside the classifier .jab filename, training date, number of training videos, and number of labeled frames.

#### Time considerations

Manual annotation of 15 test videos takes ∼8‐10 hr. Running the analysis scripts takes <30 min. The full workflow from annotation start to generating final performance metrics takes approximately one full working day.

## Alternate Protocol 3 INTEGRATION OF AN OPTOGENETICS MODULE

This protocol describes the integration of a green‐light optogenetics module with the DANCE recording hardware for circuit‐level dissection of aggression behavior. Two printed circuit boards, each carrying five green LEDs, are positioned diagonally ∼8 cm from the DANCE arena and controlled by an Arduino microcontroller. Illumination silences neurons expressing the anion channelrhodopsin GtACR1 during the 20‐min aggression recording window. The protocol assumes that DANCE aggression hardware has been assembled (Basic Protocol 1), flies have been loaded and are acclimatized (Basic Protocol 1, steps 6‐13), and video acquisition has been configured (Support Protocol 3). An annotated schematic and photograph of the complete optogenetics recording setup are shown in Figure 7—figure supplement 1 of Yadav et al. (2025; https://elifesciences.org/articles/105465/figures#fig7s1).

*NOTE*: This protocol uses *UAS‐GtACR1*, which drives expression of a green‐light‐activated anion channelrhodopsin (Govorunova et al., 2015; Mohammad et al., 2017). GtACR1 is an inhibitory channelrhodopsin that silences neuronal activity upon green light illumination. GtACR1‐mediated silencing in these experiments was achieved without dietary supplementation with all‐*trans* retinal (Schnaitmann et al., 2024).

Worked example genotype from Yadav et al. (2025): Cross *TH‐*GAL4 virgin females (Bloomington *Drosophila* Stock Center, BDSC no. 51982) with *UAS‐GtACR1* males (BDSC no. 92983; originally a gift from Gaurav Das, National Centre for Cell Science, Pune, India). Collect male progeny of the genotype *TH‐*GAL4/+; *UAS‐GtACR1*/+ for behavioral assays. Use parental control lines (*TH‐*GAL4/+ and *UAS‐GtACR1*/+ males) in the same session.

The white tablet backlight used for video recording contains spectral power within the GtACR1 excitation range and is not spectrally inert with respect to the opsin. Screen brightness was set to the minimum level that still permitted reliable tracking, and the same setting was used for the experimental genotype and both parental controls within a session. Under these conditions, no substantial change in aggression attributable to the backlight was observed.

Optogenetic silencing of dopaminergic neurons using *TH‐*GAL4 > *UAS‐GtACR1* produced a significant increase in isolation‐induced aggressive lunges across arena sizes of 13 mm, 17 mm, and 21 mm diameter, as shown in Figure 7—figure supplement 3 of Yadav et al. (2025; https://elifesciences.org/articles/105465/figures#fig7s3). Increasing arena size tends to reduce lunge frequency, though the differences were not statistically significant. Representative recordings showing the resulting increase in aggression upon dopaminergic neuron silencing are available as Figure 7—video 2 of Yadav et al. (2025; https://elifesciences.org/articles/105465/figures#fig7video2).

### Materials

High‐power green LEDs (10; 520‐540 nm, peak emission 530 nm; Lumileds, model L128‐GRN1003500000)
Printed circuit boards, two (for mounting 5 LEDs each)
Soldering kit (soldering iron, solder, flux)
LED stands or mounts
Arduino microcontroller
LED driver or current‐control module
Computer (for Arduino programming and power supply via USB)
Digital multimeter (for verifying supply voltage and per‐strip current draw)
Assembled DANCE aggression arena (Basic Protocol 1)
Electronic tablet for backlight
Smartphone camera (Android or iPhone)

1 Solder five green LEDs onto each of the two printed circuit boards, maintaining a spacing of ∼1 cm between adjacent LED positions. This creates the two LED strips that will illuminate the DANCE arena from the side.
2 Mount each of the two LED strips (five LEDs per strip) in its LED stand, positioned diagonally ∼8 cm from the behavioral chamber. Confirm that all wells of the arena fall within the combined illumination field of the two strips before proceeding.
3 Orient LEDs such that the light path crosses the arena plane as uniformly as possible. Uneven illumination will produce variable silencing efficiency across wells.
4 Connect the LED array to the Arduino microcontroller via the LED driver or current‐control module. Connect the Arduino to a computer via USB.
5 Upload Arduino control code to the microcontroller. The code should configure LED onset and duration to match the 20‐min recording window. Arduino code for DANCE optogenetics is available at https://github.com/agrawallab/DANCE.
6 Before placing flies in arenas, power up the LED array and confirm the drive parameters. Connect a multimeter in series with one LED strip, set to a DC current range of 200 mA, and confirm a current draw of ∼88 mA per strip at a supply voltage of 4.9 V. Record the measured voltage and current in the experiment log.
*IMPORTANT NOTE*: These drive parameters correspond to the hardware used to generate the silencing results reported in Yadav et al. (2025). Do not increase drive current without first validating behavioral and locomotor effects on the specific genotype in use. Verify voltage and current before every experiment.
7 Power off the LEDs. Assemble the DANCE arena with flies as described in Basic Protocol 1, steps 3‐13. Allow 5 min of acclimatization.
8 Place the arena on the backlight tablet. Position the smartphone camera above the arena for video recording. Configure recording parameters as described in Support Protocol 3.
9 Begin video recording (Support Protocol 3, step 9).
10 Immediately after starting the recording, activate the Arduino‐controlled LEDs to begin optogenetic stimulation. LED illumination and video recording should be simultaneous from the start of the 20‐min window.
11 Confirm that the LED illumination does not produce visible glare or overexposure artifacts in the recording. Review the first 30 sec of footage through the smartphone viewfinder. If the LED light source appears as a bright reflection in the arena ceiling, reposition the LED array to angle the illumination more obliquely.
12 Record for 20 min. At the end of the session, stop recording and power off the LEDs.

#### Time considerations

Initial LED soldering and PCB assembly is a one‐time setup process taking ∼20‐30 min. Verification of supply voltage and per‐strip current takes 5 min per session. Arena setup and fly loading take ∼5 min (see Basic Protocol 1). Acclimatization takes 5 min. Recording takes 20 min. Total session time after initial setup: ∼60 min.

## REAGENTS AND SOLUTIONS

### Alcojet cleaning solution (for washing arenas between experiments)

Dissolve 0.5 ml of Alcojet detergent (Alconox, cat. no. 1401‐1) in distilled water to 1000 ml to produce a 0.05% (w/v) solution. Submerge used blister‐pack arenas in the solution and wash gently. Do not scrub. Rinse thoroughly with distilled water at least three times to remove all detergent residue. Allow arenas to air‐dry completely at room temperature before recoating with Sigmacote. Avoid heat drying.

### Apple juice agar (for aggression food plates)

Prepare the day before use; plates require 8‐12 hr of drying at 25°C, 55%‐60% relative humidity, before use.

*Part A*: Combine 300 ml of commercial apple juice (no added sugars or preservatives) and 9 g agar (HiMedia, cat. no. GRM026) in a heat‐resistant beaker. Heat in a microwave or on a hot plate, stirring intermittently with a steel spatula, until the agar is fully dissolved and the mixture reaches a rolling boil.

*Part B*: Dissolve 10 g of sucrose (HiMedia, cat. no. GRM601) in 100 ml of commercial apple juice (no added sugars or preservatives) at room temperature.

Add Part B to the hot Part A mixture and stir thoroughly to combine.

*Pouring and drying*: Allow the mixture to cool to ∼50°C before pouring onto acrylic food plates on a level surface. Allow to solidify for 10‐15 min. Cover loosely with cheesecloth and allow to dry at 25°C and 55%‐60% relative humidity for 8‐12 hr before use. At ambient humidity >60% relative humidity, extend drying time to up to 16 hr.

*Storage*: Store prepared food plates covered with cling film for up to 3 days at 4°C. Allow refrigerated plates to equilibrate to room temperature for at least 30 min before use. Do not use plates if condensation has formed on the surface.

Final composition: 2.25% (w/v) agar in commercial apple juice with 2.5% (w/v) sucrose; total volume: 400 ml, sufficient for 16 standard 2‐mm acrylic food plates.

### Sigmacote working solution

Sigmacote (Sigma‐Aldrich, cat. no. SL2) is used directly from the bottle without dilution. Apply to arena walls and ceilings using cotton swabs. Allow coated arenas to dry overnight (minimum 8 hr) at room temperature before use.

*CAUTION*: Sigmacote contains chlorotrimethylsilane in a heptane/xylene carrier. It is flammable and a respiratory irritant. All applications must be performed in a fume hood. Store at room temperature away from heat sources.

## COMMENTARY

### Background Information

DANCE was benchmarked against CADABRA (Dankert et al., 2009) and MateBook (Ribeiro et al., 2018) in Yadav et al. (2025), using manual ground‐truth annotation as the reference, and matched or exceeded the corresponding rule‐based method on precision, recall, and *F*_1_ across both the aggression and courtship classifiers, at a fraction of the hardware cost of conventional machine‐vision setups. Readers seeking the full benchmarking data are directed to that study.

### Critical Parameters

#### Social isolation duration and housing conditions

The duration of pre‐assay single housing is the most important biological variable affecting aggression levels in male *Drosophila* and must be controlled precisely. In the *eLife* validation study (Yadav et al., 2025), Canton‐S males were collected within 24 hr of eclosion and housed individually (1 male per vial, 70 mm × 10 mm) for exactly 6 days prior to the aggression assay. Single‐housed (SH) males show substantially elevated lunge frequencies relative to group‐housed (GH) males across the full range of aggression levels. Reducing isolation to fewer than 4 days will markedly reduce aggression and push most dyads into the low‐aggressive category. Extending isolation beyond 8 days does not reliably increase aggression further and may introduce variability due to age‐related changes in behavior. For courtship experiments, males were also individually housed for 6 days. All assays were performed at 25°C and 65% relative humidity.

#### Zeitgeber time

Aggression in *Drosophila* is strongly gated by circadian time. Aggression assays must be performed during ZT0‐ZT2.5 (the first 2.5 hr of the light phase), which corresponds to the morning activity peak. Courtship assays should be performed during ZT0‐ZT3 or ZT9‐ZT12. Performing assays outside these windows will reduce behavioral output and increase inter‐dyad variability. Record the clock time at which each assay was started and verify that it falls within the appropriate ZT window before including the resulting data in analysis.

#### Food plate moisture

The moisture content of the apple‐juice agar food plate at the time of assay directly affects fly locomotion and behavior. Excess surface moisture causes increased grooming, reduced locomotion, and suppressed aggression. Food plates should be dried at 25°C and 55%‐60% relative humidity for 8‐12 hr before use. At high ambient humidity (>60% relative humidity), drying may require up to 16 hr. The surface should appear matte and non‐reflective. A shiny surface indicates excess moisture; discard the plate.

#### Sigmacote coating uniformity

Incomplete Sigmacote coating causes flies to climb arena walls and ceiling, which disrupts inter‐fly interactions and produces artifactually low behavioral scores. Before each experiment, visually inspect coated arenas under bright light to confirm uniform coating. Verify effectiveness by placing a single fly in a coated well for 1‐2 min before loading experimental flies. A correctly coated arena will keep the fly on the food surface. Re‐coat arenas where wall‐climbing is observed. Sigmacote coating must be performed in a fume hood. DANCE applies Sigmacote to both the walls and the ceiling in place of the Fluon wall coating used in conventional multiwell chambers (Dierick, 2007; Dankert et al., 2009), because Fluon dries to an opaque film that would obscure the ceiling through which recording is made.

#### FlyTracker calibration accuracy

The accuracy of all downstream behavioral quantification depends on correct FlyTracker calibration. The ruler length must match the actual arena diameter used (13 mm for the standard aggression arena; 11 mm for the standard courtship arena). Incorrect ruler length will cause all distance‐ and velocity‐based features to be systematically miscalculated, producing incorrect JAABA classification even when tracking appears visually accurate. After calibration, inspect the background model. The Bg Mean image should show a clean, uniformly lit background with no fly‐shaped artifacts. If fly shapes are visible in the background model, recalibrate using a video segment where both flies are moving throughout.

#### JAABA classifier score thresholds and behavioral index calculation

JAABA outputs a per‐frame probability score for each behavior. Do not modify classifier score thresholds unless generating a custom classifier (Alternate Protocol 1). For the lunge classifier, output is reported as total lunge count per 20‐min session, not as a behavioral index. These calculations are performed automatically by the DANCE analysis scripts. For courtship classifiers, the behavioral index is calculated as the proportion of total recording frames classified as the behavior, using the courtship_bout_num_extractor.py script (see Basic Protocol 3, steps 12b and 13b).

#### LED illumination for optogenetics (Alternate Protocol 3)

Silencing efficiency depends on reproducing the LED drive conditions and geometry specified in Alternate Protocol 3: a 4.9‐V supply delivering ∼88 mA per strip of five high‐power green LEDs (520‐540 nm, peak emission 530 nm; Lumileds L128‐GRN1003500000), with each of the two strips positioned diagonally ∼8 cm from the behavioral chamber. Verify voltage and per‐strip current before each experiment using a multimeter connected in series with the strip. Changes to drive current, LED count, or working distance alter the delivered illumination and may produce inconsistent silencing.

#### Classifier transferability across genetic backgrounds

The lunge classifier was trained and validated on wild‐type Canton‐S, wild‐type Berlin, and a GAL4 driver line outcrossed into the Berlin background. The five courtship classifiers were trained predominantly on wild‐type Canton‐S flies. JAABA per‐frame features combine tracked body and wing geometry with kinematic measures such as velocity, angular position, and distance to the other fly or the arena wall, so classifier performance is expected to hold across genotypes with comparable movement dynamics and body proportions to these training backgrounds, and to degrade for genotypes with pronounced differences in either, for example other *Drosophila* species, mutants with substantial wing or leg defects, or flies subjected to manipulations that markedly alter locomotor activity. Users working with such a genotype, or with different recording conditions or arena geometry, should validate the distributed classifiers on their own background using Alternate Protocol 2 before generating experimental data. Where the resulting *F*_1_ score falls below 0.70, the acceptance threshold already specified for newly trained classifiers, retrain on the new background using Alternate Protocol 1.

#### Statistical analysis and unit of replication

The unit of replication in all DANCE experiments is the dyad (pair of flies), not the individual fly. Report *n* as the number of dyads per condition. Behavioral indices and lunge counts are typically non‐normally distributed and should be compared using non‐parametric tests (Kruskal‐Wallis ANOVA with Dunn's post‐hoc test for multiple comparisons, or Mann‐Whitney *U* test for pairwise comparisons). Statistical analysis was performed in GraphPad Prism 8 in the eLife validation study.

### Troubleshooting

Common problems encountered during this protocol, their likely causes, and recommended solutions are listed in Table 3.

**Table 3:** Troubleshooting Guide for the DANCE Protocol

Problem	Protocol step	Possible cause	Solution
Arena circle is misaligned or incorrectly sized during video segmentation	BP3, steps 4‐9	Low arena‐to‐background contrast in source video; arena partially obstructed	Inspect source video; recheck lighting setup (SP3, steps 1‐4); re‐record if contrast is insufficient.
FlyTracker fails to open after typing tracker in MATLAB	SP4, step 4	FlyTracker or JAABA not added to MATLAB path; MATLAB restarted since path was saved	Re‐add FlyTracker and JAABA folders via Home, then Set Path, then Add with Subfolders; save path
Background model shows fly‐shaped artifacts	SP4, step 9	Background computed over frames containing stationary flies	Rerun calibration using a different video segment where both flies are moving throughout
Chamber detection produces incorrect boundaries	SP4, steps 11 and 12	Arena not centered in cropped video; arena dimensions differ from expected	Select Manually Set Chambers; drag boundary to match arena edge; verify ruler length matches arena diameter
Flies climb arena walls during assay	SP2 and BP1	Incomplete or aged Sigmacote coating	Re‐coat arenas in fume hood; verify coating by single‐fly test before experiment; replace Sigmacote if older than 12 months
JAABA produces empty score files	SP5, steps 9‐12	Frame rate mismatch between new videos and classifier training data; wrong .jab file loaded	Verify all videos are 30 fps (SP3, step 6); confirm correct .jab filename was selected; re‐run classification
Lunge_Analyzer_indiC.py returns .csv file with fewer rows than expected	BP3, step 12a	Score files missing for one or more chambers due to incomplete JAABA classification	Return to SP5, step 11; confirm that scores_Lunge.mat is present in every _JAABA/ folder; re‐classify missing chambers
Lunge counts are implausibly low across all dyads (fewer than 5 lunges per 20 min)	BP3 and Commentary	Insufficient social isolation; assay performed outside ZT0 to ZT2.5; excess food plate moisture	Verify 6‐day single housing; check clock time of assay; inspect food plate surface; repeat with freshly prepared food plates
Courtship identity assignment inverted (female labeled as male)	BP3, step 16	Male did not perform wing extension; mislabeled loading holes; wrong genotype	Inspect raw video to confirm male identity; verify fly genotype; verify loading procedure (BP2, steps 10 and 11)
High inter‐dyad variability in lunge counts within same genotype	Commentary	Variation in isolation duration; variation in ZT of assay; fly age heterogeneity	Standardize collection to a single day post‐eclosion; confirm all flies are 6‐day isolated; restrict assay window to ZT0‐ZT2.5
Identity swaps cannot be corrected in FlyTracker Visualizer	SP4, steps 24‐27	Excessive fly overlap throughout the video; poor contrast; recording at non‐standard fps	Check recording fps and resolution (SP3, step 6); if flies overlap for more than 10% of the recording, consider excluding the video
Courtship output .csv file contains NaN values for one or more classifiers	BP3, step 16	Corresponding classifier was not loaded in JAABA; score file renamed incorrectly	Return to SP5, step 8b, and verify that all five courtship .jab files were loaded and classified.
Classifier performance appears poor on a genotype outside the training backgrounds	Commentary	Genotype differs substantially from the training backgrounds in body proportions or movement dynamics	Validate on the new genotype using AP2, with *F*_1_ of ≥0.70 as the acceptance criterion; retrain using AP1 if the criterion is not met

### Understanding Results

#### Expected output for aggression experiments

Users should expect to observe higher lunge counts in SH males compared to GH males. This is demonstrated for wild‐type Canton‐S males in Yadav et al. (2025; Fig. 7N and O). If lunge counts fall systematically below the expected range in comparably isolated wild‐type males, check isolation duration, assay timing, food plate moisture, and arena coating before concluding that an experimental manipulation reduced aggression. Representative visualization and quantification of lunge events from a single DANCE aggression recording are shown in Figure 3. Representative aggression and courtship behaviors recorded using DANCE hardware are shown in Figure 7—video 1 of Yadav et al. (2025; https://elifesciences.org/articles/105465/figures#fig7video1).

**Figure 3:**
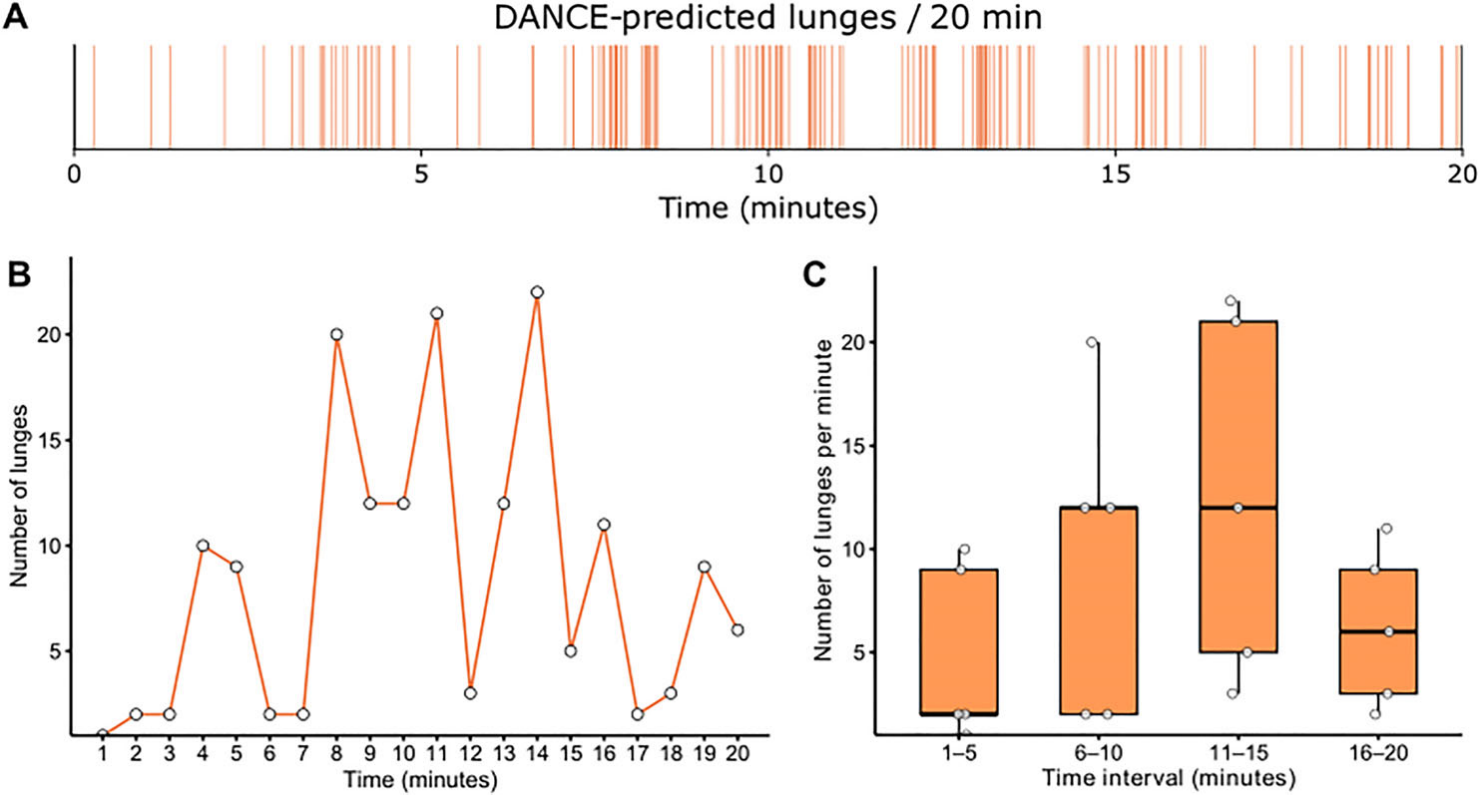
Visualization and quantification of *Drosophila* aggressive lunge events detected using DANCE. (**A**) Raster plot showing the temporal distribution of DANCE‐predicted lunges during a 20‐min aggression assay. Each vertical line represents a single lunge event identified by the lunge classifier throughout the recording. (**B**) Time‐series plot showing minute‐by‐minute quantification of lunge behavior. The number of lunges detected during each 1‐min interval is plotted across the 20‐min recording period. (**C**) Summary of lunge frequency across four consecutive 5‐min intervals. Box and whisker plots show the distribution of lunge counts per minute within each interval. Data were generated using a single‐housed TH‐GAL4〉UAS‐GtACR1 male tested in the 13‐mm DANCE aggression arena under green light illumination under the experimental conditions described in Yadav et al. (2025; Fig. 7R). Lunges were detected using the DANCE lunge classifier.

#### Expected output for courtship experiments

Users should expect to observe higher courtship indices in SH males than in GH males for wing extension, attempted copulation, and circling. Following does not consistently differ between housing conditions. These patterns are demonstrated for wild‐type Canton‐S males paired with decapitated virgin females in Yadav et al. (2025; Figs. 7D, F, H, and J). Decapitated virgin females were also used in classifier training and validation to enrich rare behaviors such as attempted copulation and circling (Figs. 3‐5 of Yadav et al., 2025). Results obtained with decapitated virgin females and with mated females should not be pooled. If courtship indices fall systematically below the expected range in comparably housed wild‐type males, check isolation duration, assay timing, female preparation, and arena coating before concluding that an experimental manipulation reduced courtship. Representative visualization and quantification of courtship behaviors from a single DANCE courtship recording are shown in Figure 4. The 15‐min recording window captures the occurrence and latency of copulation but not its duration to completion; studies requiring copulation duration should extend the recording until copulation has ended. Representative courtship behaviors recorded using DANCE hardware are shown in Figure 7—video 1 of Yadav et al. (2025; https://elifesciences.org/articles/105465/figures#fig7video1).

**Figure 4:**
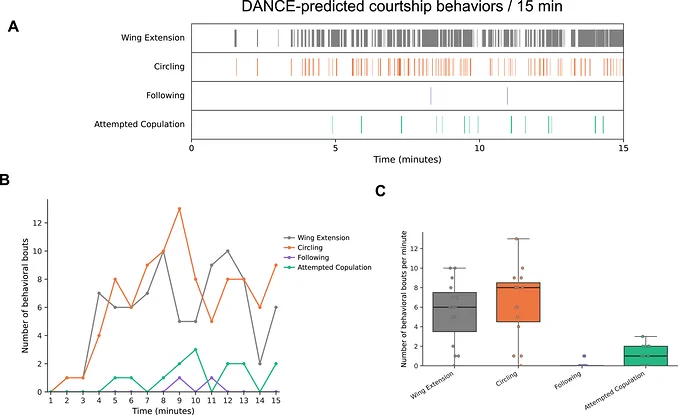
Visualization and quantification of DANCE‐predicted courtship behaviors. (**A**) Raster plot showing the occurrence of four courtship behaviors detected by DANCE (wing extension, circling, following, and attempted copulation) in interactions between a male fly and a decapitated virgin female fly. Each bar represents a behavioral bout, with bar length showing bout duration, illustrating the temporal dynamics of courtship throughout the assay. (**B**) Time‐series plot showing minute‐by‐minute quantification of behavioral bouts detected during the 15‐min recording. The line graph depicts the frequencies of wing extension, circling, following, and attempted copulation. (**C**) Box and whisker plots show the distribution of per‐minute bout counts across the 15‐min recording for each courtship behavior. Data were generated from a single recording of a Canton‐S male paired with a decapitated virgin female, tested in the DANCE courtship assay as part of the classifier validation set in Yadav et al. (2025). Behaviors were detected using the DANCE courtship classifiers.

#### When to exclude a video from analysis

Exclude a video from analysis if any of the following conditions are present: (a) one or both flies are not visible for >10% of the recording duration; (b) more than five uncorrectable identity swaps are present in the FlyTracker tracking output; (c) one fly died or became immobile during recording; or (d) the arena seal failed and one fly escaped. Record all exclusions in the experimental log with reason. Do not exclude videos based on low behavioral output alone.

### Time Considerations

The following schedule describes the recommended timeline for a standard 8‐dyad aggression experiment from arena preparation through final data output.

Day 1: Collect males within 24 hr of eclosion and house individually (Support Protocol 1).

Day 6: Prepare and coat arenas with Sigmacote (Support Protocol 2, Part B). Allow to dry overnight at room temperature. This step cannot be compressed. The coating requires a minimum of 8 hr to cure, and coated arenas cannot be used the same day they are prepared. Prepare apple‐juice agar food plates (Support Protocol 2, Part A) and allow to dry at 25°C and 55%‐60% relative humidity for 8‐12 hr. Prepare the backlight tablet by opening the white‐screen light application. Verify smartphone recording settings (Support Protocol 3).

Day 7, morning (ZT0‐ZT2.5): Assemble DANCE hardware (Basic Protocol 1, ∼5 min for 8 wells). Load flies by mouth aspirator (∼3 min for 8 wells; see Basic Protocol 1). Allow 5 min of acclimatization per arena. Record 20‐min aggression videos. Total morning experimental time: ∼90 min.

Day 7, late morning: Transfer videos from smartphone to computer. Organize into a date folder. Run Auto_Cropper.py (∼5 min). Run FlyTracker tracking on all eight segmented videos (∼15 min). Inspect and correct identity swaps in FlyTracker Visualizer (∼20 min). Run JAABA lunge classification (∼10 min). Run Lunge_Analyzer_indiC.py (<1 min). Total computational time: ∼50 min.

Total elapsed time from start of hardware assembly to analyzed .csv output: ∼3 hr for a standard 8‐dyad aggression experiment. For courtship experiments, substitute the 15‐min recording window and add an additional ∼20 min for JAABA classification with all five courtship classifiers.

### Author Contributions

**R. Sai Prathap Yadav**: Methodology; investigation; data curation; software; formal analysis; validation; visualization; writing—original draft; writing—review and editing. **Paulami Dey**: Methodology; investigation; data curation; software; formal analysis; validation; visualization; writing—review and editing. **Pavan Agrawal**: Conceptualization; resources; supervision; funding acquisition; visualization; methodology; writing—original draft; project administration; writing—review and editing.

### Conflict of Interest

R.S.P.Y. and P.A. are named inventors on an Indian patent application related to the work described herein, entitled “Device for Measuring Complex Social Behaviors in Small Insects” (application no. 202441072884). P.D. declares no conflict of interest.

## Data Availability

All custom analysis scripts, DANCE behavioral classifiers, and documentation used in this protocol are publicly available in the GitHub repository at https://github.com/agrawallab/DANCE. Some scripts were generated and debugged with assistance from ChatGPT or Claude (Anthropic) and were subsequently reviewed and experimentally verified by the authors to ensure accuracy and functionality. Detailed instructions are provided to facilitate implementation and reproducibility.

## References

[cpz170511-bib-0001] Chowdhury, B. , Wang, M. , Gnerer, J. P. , & Dierick, H. A. (2021). The divider assay is a high‐throughput pipeline for aggression analysis in Drosophila. Communications Biology, 4, 85. 10.1038/s42003-020-01617-6 PMC7815768 33469118

[cpz170511-bib-0002] Dankert, H. , Wang, L. , Hoopfer, E. D. , Anderson, D. J. , & Perona, P. (2009). Automated monitoring and analysis of social behavior in Drosophila. Nature Methods, 6, 297–303. 10.1038/nmeth.1310 PMC2679418 19270697

[cpz170511-bib-0003] Dierick, H. A. (2007). A method for quantifying aggression in male *Drosophila melanogaster* . Nature Protocols, 2, 2712–2718. 10.1038/nprot.2007.404 18007606

[cpz170511-bib-0004] Eyjolfsdottir, E. , Branson, S. , Burgos‐Artizzu, X. P. , Hoopfer, E. D. , Schor, J. , Anderson, D. J. , & Perona, P. (2014). Detecting social actions of fruit flies. In Lecture notes in computer science Vol. 8690 (pp. 772–787). Springer International Publishing.

[cpz170511-bib-0005] Govorunova, E. G. , Sineshchekov, O. A. , Janz, R. , Liu, X. , & Spudich, J. L. (2015). Natural light‐gated anion channels: A family of microbial rhodopsins for advanced optogenetics. Science, 349, 647–650. 10.1126/science.aaa7484 PMC4764398 26113638

[cpz170511-bib-0006] Kabra, M. , Robie, A. A. , Rivera‐Alba, M. , Branson, S. , & Branson, K. (2013). JAABA: Interactive machine learning for automatic annotation of animal behavior. Nature Methods, 10, 64–67. 10.1038/nmeth.2281 23202433

[cpz170511-bib-0007] Leng, X. , Wohl, M. , Ishii, K. , Nayak, P. , & Asahina, K. (2020). Quantifying influence of human choice on the automated detection of Drosophila behavior by a supervised machine learning algorithm. PLOS ONE, 15, e0241696. 10.1371/journal.pone.0241696 PMC7743940 33326445

[cpz170511-bib-0008] Mohammad, F. , Stewart, J. C. , Ott, S. , Chlebikova, K. , Chua, J. Y. , Koh, T. W. , Ho, J. , & Claridge‐Chang, A. (2017). Optogenetic inhibition of behavior with anion channelrhodopsins. Nature Methods, 14, 271–274. 10.1038/nmeth.4148 28114289

[cpz170511-bib-0009] Ribeiro, I. M. A. , Drews, M. , Bahl, A. , Machacek, C. , Borst, A. , & Dickson, B. J. (2018). Visual projection neurons mediating directed courtship in Drosophila. Cell, 174, 607–621.e18. 10.1016/j.cell.2018.06.020 30033367

[cpz170511-bib-0010] Schnaitmann, C. , Pagni, M. , Meyer, P. B. , Steinhoff, L. , Oberhauser, V. , & Reiff, D. F. (2024). Horizontal‐cell like Dm9 neurons in Drosophila modulate photoreceptor output to supply multiple functions in early visual processing. Frontiers in Molecular Neuroscience, 17, 1347540. 10.3389/fnmol.2024.1347540 PMC11133737 38813436

[cpz170511-bib-0011] Simon, J. C. , & Heberlein, U. (2020). Social hierarchy is established and maintained with distinct acts of aggression in male *Drosophila melanogaster* . The Journal of Experimental Biology, 223, jeb232439. 10.1242/jeb.232439 PMC7774903 33268534

[cpz170511-bib-0012] Yadav, R. S. P. , Dey, P. , Ansari, F. , Kottat, T. , Vasam, M. , Prabhu, P. P. , Ayyangar, S. , Swathi, B. S. , Prabhu, K. , Ghosh, M. , & Agrawal, P. (2025). DANCE provides an open‐source and low‐cost approach to quantify aggression and courtship in Drosophila. eLife, 14, RP105465. 10.7554/eLife.105465 PMC12747525 41459640

